# Sustainable agriculture through seaweed biostimulants: a two-year study demonstrates yield enhancement in pepper and eggplant

**DOI:** 10.3389/fpls.2025.1655340

**Published:** 2025-09-08

**Authors:** Nikola Staykov, Aakansha Kanojia, Rafe Lyall, Valentina Ivanova, Saleh Alseekh, Veselin Petrov, Tsanko Gechev

**Affiliations:** ^1^ Department Molecular Stress Physiology, Center of Plant Systems Biology and Biotechnology, Plovdiv, Bulgaria; ^2^ Department Genetics of Crop Metabolism, Max Planck Institute of Molecular Plant Physiology, Potsdam, Germany; ^3^ Department of Plant Physiology, Biochemistry and Genetics, Agricultural University, Plovdiv, Bulgaria; ^4^ Department of Molecular Biology, Plovdiv University “Paisii Hilendarski”, Plovdiv, Bulgaria

**Keywords:** sustainable agriculture, biostimulant, crop resilience, yield enhancement, omics

## Abstract

Global climate change and unsustainable agricultural practices have intensified the need for eco-friendly strategies to improve crop resilience and productivity. This study evaluates the efficacy of a seaweed-based biostimulant derived from Ascophyllum nodosum extract (ANE) in enhancing yield-related traits in pepper (*Capsicum annuum*) and eggplant (*Solanum melongena*) under open-field conditions over two consecutive years. Foliar applications of ANE were performed during early flowering stages, and plant performance was assessed using yield measurements, metabolomics, transcriptomics, and elemental analyses. ANE application significantly increased total fruit yield, primarily through higher fruit numbers per plant and, to a lesser extent, increased fruit size/weight, with hints to these effects traced back to earlier developmental stages. Metabolomic analyses revealed that ANE treatment modulated primary metabolism, enhancing sugar and amino acid levels, nitrogen assimilation, and osmoprotection, which together supported improved fruit set and development. Transcriptomic profiling demonstrated consistent gene expression changes in pathways related to cell wall modification, stress response, and carbohydrate metabolism. Elemental analysis indicated a general nutrient dilution effect due to increased biomass, with the notable exception of magnesium, which was enriched in ANE-treated pepper fruits. These findings highlight the potential of ANE as a sustainable agricultural input to improve yield and quality in vegetable crops while supporting environmentally responsible farming practices.

## 1 Introduction

Global climate change poses a significant threat to agricultural production, impacting food security worldwide (Saleem et al., 2024). Unpredictable weather patterns, and extreme climatic events have led to considerable yield losses in major crops, including vegetables (Lesk et al., 2016). Abiotic stressors such as heat, drought, and soil salinity have reduced overall crop productivity, making it crucial to develop sustainable agricultural strategies (Bailey-Serres et al., 2019). Moreover, the extensive use of chemical fertilizers and pesticides has further degraded soil health and posed risks to human health and the environment (Khatun et al., 2022). Farmers are facing economic losses due to declining yields and increased production costs, necessitating the adoption of eco-friendly biostimulants to enhance crop resilience and productivity.

Biostimulants have emerged as a promising approach to mitigate abiotic stress effects and improve crop yields in an environmentally sustainable manner (Di Sario et al., 2025; Mandal et al., 2023). They include natural substances that enhance plant growth, nutrient uptake, and stress tolerance without being direct nutrient sources (du Jardin, 2015; Liang et al., 2023; Han et al., 2024). Among the various biostimulants, seaweed extracts have received significant attention due to their beneficial effects on plant growth, development, and resistance to environmental stress (Kanojia et al., 2024; Raja and Vidya, 2023; Ali et al., 2021; El Boukhari et al., 2020). Seaweed-based biostimulants derived from marine macroalgae are rich in bioactive compounds such as polysaccharides, phytohormones, and secondary metabolites that stimulate physiological and biochemical processes in plants (Mughunth et al., 2024; El-Beltagi et al., 2022). These extracts have been shown to exert their beneficial effects through various mechanisms. For instance, they stimulate root development (Arioli et al., 2024), improve nutrient uptake like Ca, N, P, K, S, Na, Mg (Rathore et al., 2009; Crouch et al., 1990) and increase photosynthetic efficiency (Yao et al., 2020; Ali et al., 2021). Additionally, seaweed extracts can improve soil structure and microbial activity when applied trough fertigation, leading to better water retention and nutrient availability (Mughunth et al., 2024; Najafi Vafa et al., 2022). Studies have reported that they induce significant yield increases in various crops, including a 15.17% average increase across multiple crop types, and a 4.6–6.9% increase in tomato yields, attributed to enhanced photosynthetic capacity and improved fruit quality (Hernández-Herrera et al., 2022; Yao et al., 2020; Rathore et al., 2009). Moreover, seaweed extracts have been found to enhance plants tolerance to abiotic stresses such as drought and salinity and the related to them oxidative stress, further contributing to improved crop performance (Staykov et al., 2020; Krid et al., 2023; Kanojia et al., 2024).

Pepper (*Capsicum annuum*) and eggplant (*Solanum melongena*) are economically significant vegetable crops, widely cultivated in Europe and other regions. However, both crops are highly sensitive to abiotic stress, which results in substantial yield losses. In Europe, pepper and eggplant production faces challenges due to fluctuating temperatures, drought episodes, and soil degradation. The implementation of sustainable agricultural practices, such as the use of biostimulants, can potentially enhance their productivity and improve farmers’ income.

The goal of this study was to investigate the effects of a seaweed-based biostimulant derived from an *Ascophyllum nodosum* extract (ANE), on the yield-related properties of pepper and eggplant under field conditions. The experiment was conducted over two consecutive years to evaluate the consistency of ANE impact on total yield and yield parameters. The plants were treated with foliar applications of ANE at critical flowering stages, and their physiological and molecular responses were assessed. Additionally, metallomic, transcriptomic and metabolomic analyses were performed to elucidate the systems biology mechanisms underlying the observed effects. The study highlights the potential of seaweed-based biostimulants as a sustainable additive to conventional fertilizers, promoting higher agricultural productivity while minimizing environmental impact. Our findings could provide valuable insights for future applications in commercial vegetable production and climate-resilient farming practices.

## 2 Results

Plants were sprayed during flowering to prime the developing fruits with bioactive substances as early as possible, namely at the first flowering bud and at the Full bloom stage (8–15 days later depending on climatic conditions). Control groups were sprayed with water only. The concentration of ANE was set according to the recommendations of the manufacturer. Samples were taken at predetermined developmental stages and physiological and molecular biological properties of the tested plant groups were subsequently examined.

### 2.1 Fruit growth, fruit size distribution and yield

Fruits were examined during development at two time points – once when the majority of fruits were at the Cell Division (CD) stage (measurement one, M1) and again when most fruits were at the Fully Expanded (FE) stage (measurement two, M2) (see Methods 4.1). All standing fruits were counted and measured by width, and thereafter whole fruit and fully-grown leaf samples were collected from both the timepoints and flash-frozen for further molecular examinations. After mass maturation, mature fruits were harvested, counted and weighed for yield determination.

#### 2.1.1 ANE application increased total fruit yield during the first year of harvest

**Figure 1:**
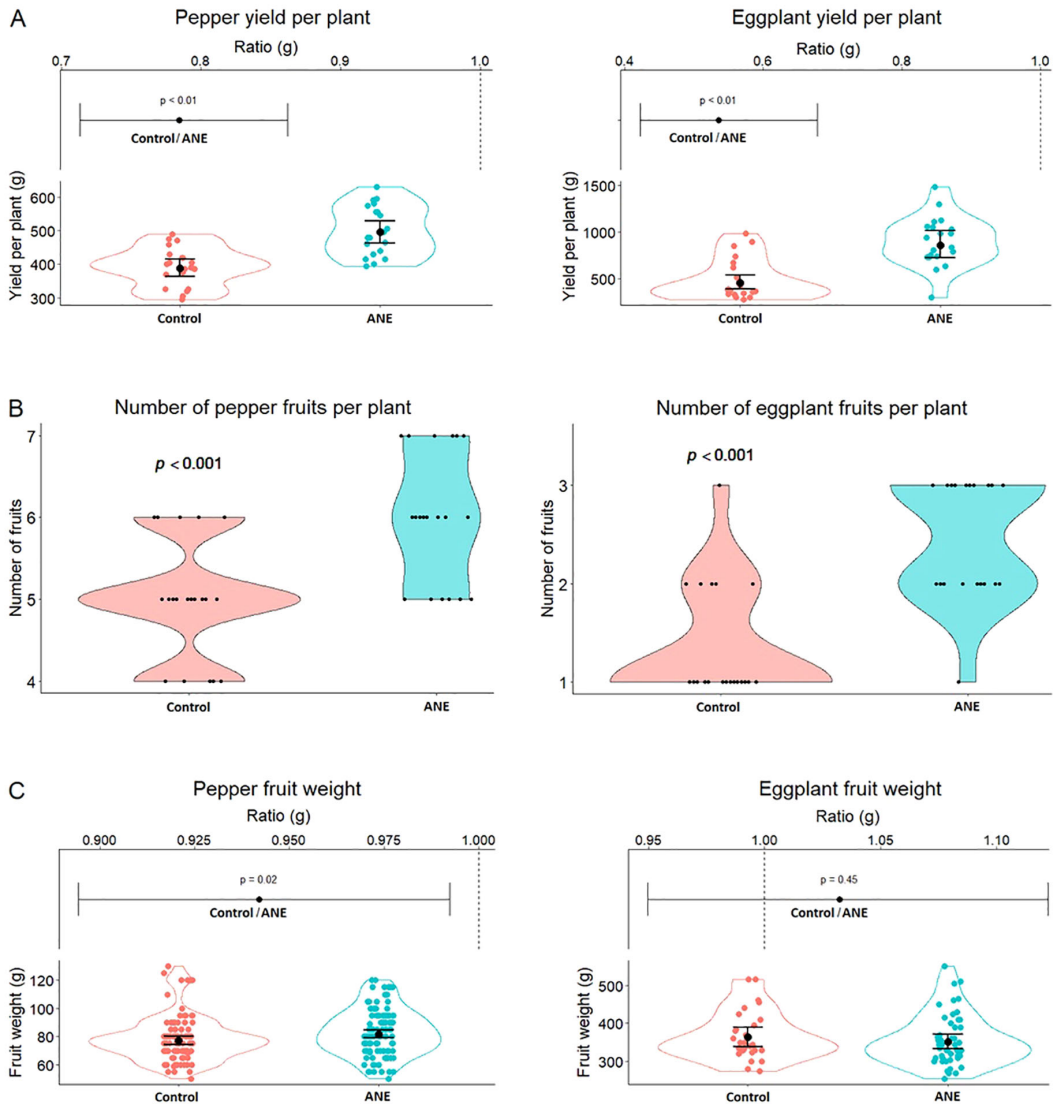
An increase in yield and fruit number (per plant) in pepper and eggplant during the first experimental year after double treatment with ANE-extract. **(A)** Total yield per plant (in grams) of ANE-treated and non-treated (Control) groups. **(B)** Total fruit number per plant of treated and non-treated groups. **(C)** Plots of individual fruit weight (in grams). For **(A, C)**: Lower graphs - scatter plots into violin plots, upper graphs: the ratio between the values of the two treatments. For All: Each group consists of n = 20 plants. All data were plotted, using a mixed linear model (as described in Materials and Methods), confidence intervals are used.

The post-harvest measurements in the first year (Year 1, **Figure 1**) showed that ANE priming resulted in several improved trait parameters in both pepper and eggplant, including significantly higher yield in both crops (**Figure 1A**), where the yield of pepper and eggplant fruits increased with 28% and 81% respectively. This trend was primarily driven by increased fruit number in ANE-treated plants, with less pronounced effect in pepper and more pronounced in eggplant, increasing with 20% and 81% respectively (**Figure 1B**). The average ANE-primed fruit weight per plant of pepper indicated a slight increase (6%), something which was not observed in eggplant (**Figure 1C**, *p* = 0.02).

**Figure 2:**
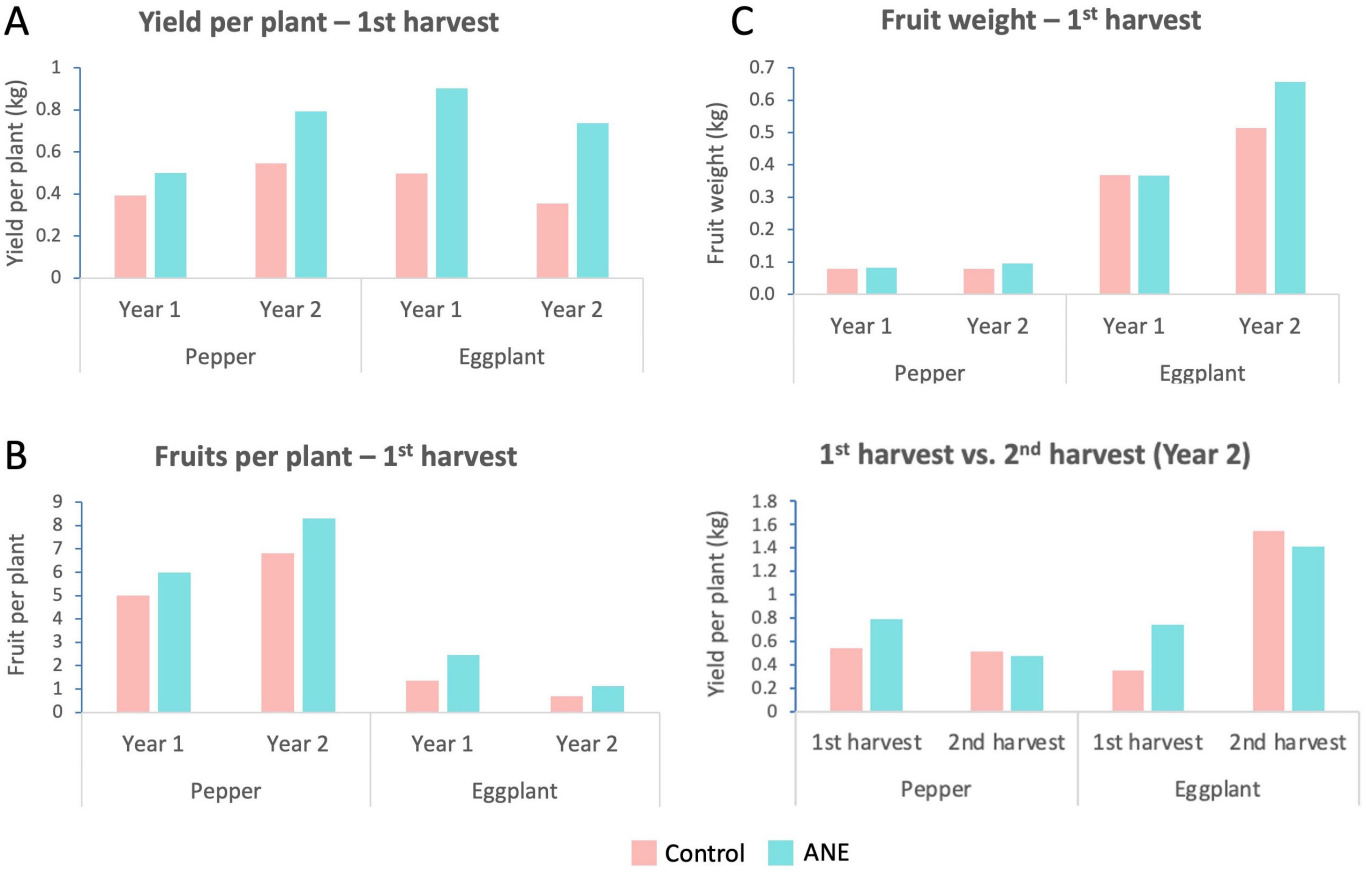
Comparison of the yield and yield-related characteristics at different time points. Column charts of average values per plant of ANE-treated and non-treated (Control) groups for both crops (pepper and eggplant). **(A)** The yield at first harvest, plotted separately for Year1 and Year 2 shows a persistent increase for the ANE-treated groups. **(B)** The fruit number at first harvest always increases upon ANE application for both crops. **(C)** Fruit weight has a tendency to increase, which is not always consistent. **(D)** The first harvest of the second year is much larger when both species are primed with ANE, while the second harvest of the primed plants is slightly lower. Graphs are compiled with data from one single growing season. Every repetition consists of n = 20 (first year) or n = 16 (second year) plants. The data points represent means calculated by dividing the values for the entire measured population to the number of fruits, not by assessing each fruit individually.

These measurements were repeated in the following growing season to independently confirm the effect of ANE treatment on yield characteristics. Overall, the additional repetition of this experiment shows the same, but even stronger trend of harvest yield increases upon ANE priming, where pepper and eggplant yields rose with 46% and 108% respectively (**Figure 2A**). The fruit number of ANE-treated plants was also elevated with similar rates – for pepper and eggplant – 22% and 64% respectively (**Figure 2B**). The average fruit weight per plant though, this time increased more significantly upon ANE-priming, reaching 20% and 27% for pepper and eggplant respectively (**Figure 2C**). Additionally, we were able to collect and analyze a second harvest (5–8 days after first harvest) for both crops. The data show a trend for a small decrease in the yield of the second harvest for both species (**Figure 2D**, which however does not interfere with the general trend of ANE-mediated increase of the total yield (**Supplementary Table S1**).

#### 2.1.2 Impact of ANE application on fruit number and size throughout development

**Figure 3:**
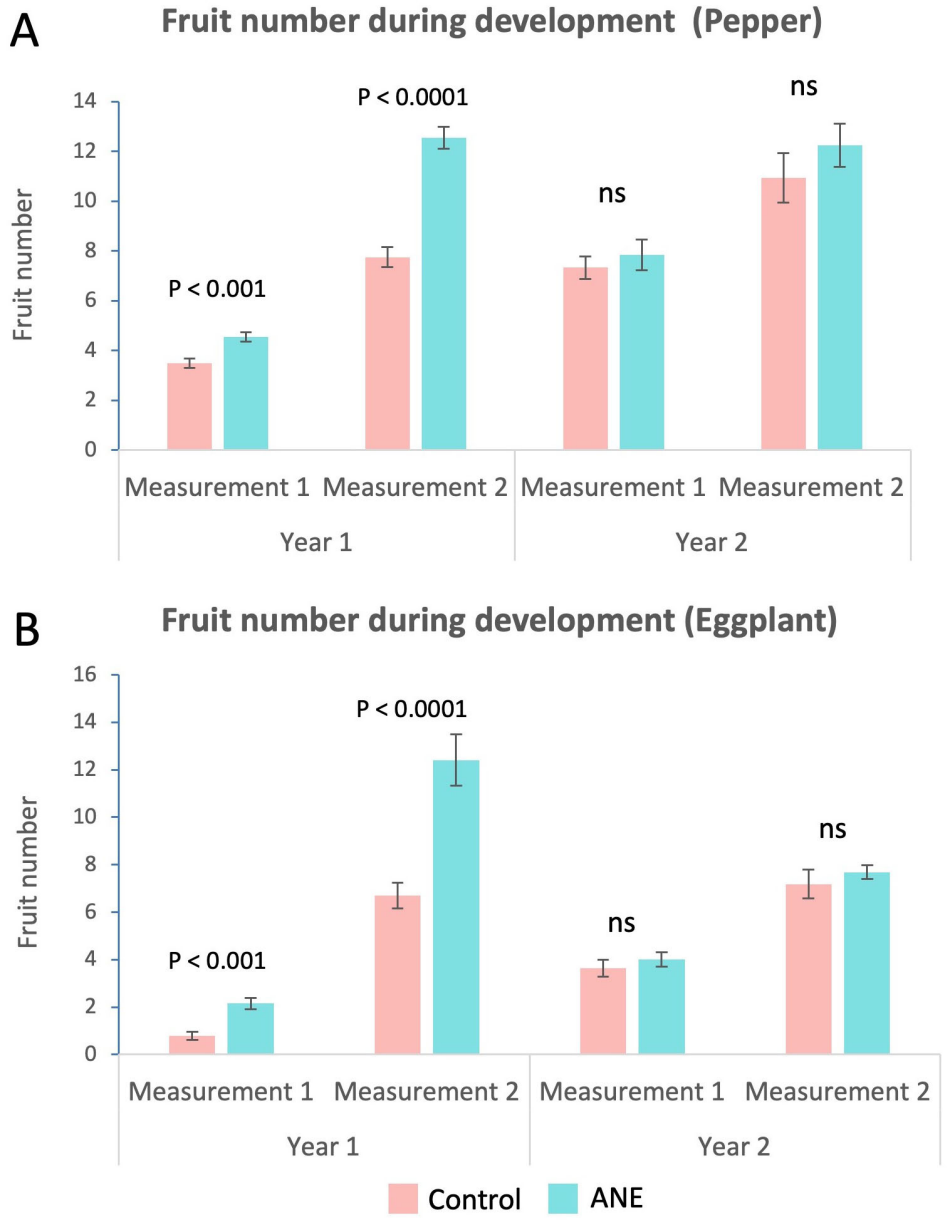
Changes in fruit number during development. Fruit number was assessed twice a year during development – when the first fruit set was at the Cell division stage (M1), and when the first fruit set was at the Fully expanded cells stage (M2). Column charts of average fruit number per plant of ANE-treated and non-treated (Control) groups. **(A)** Increase in the average fruit number of ANE-primed groups in pepper. The effect is much stronger and significant for the first year. **(B)** Increase in the average fruit number of ANE-primed groups in eggplant. A similarly stronger and significant effect is visible for the first year only. Graphs are compiled with data from two growing seasons. Every repetition consists of n = 20 (first year) or n = 16 (second year) plants. Error bars – Standard Error. Statistical significance, relative to controls is indicated above each column pair and is determined by using Student’s t-test (ns, not significant).

In both species, the number of fruits from ANE-treated plants was higher, especially at the later FE stage (**Figure 3**) and this trend kept similar values also during harvest (**Figure 2B**). However, strong yearly variation was observed and the increase was much stronger and statistically significant during the first year, while fading away during the second year (**Figure 3**).

**Figure 4:**
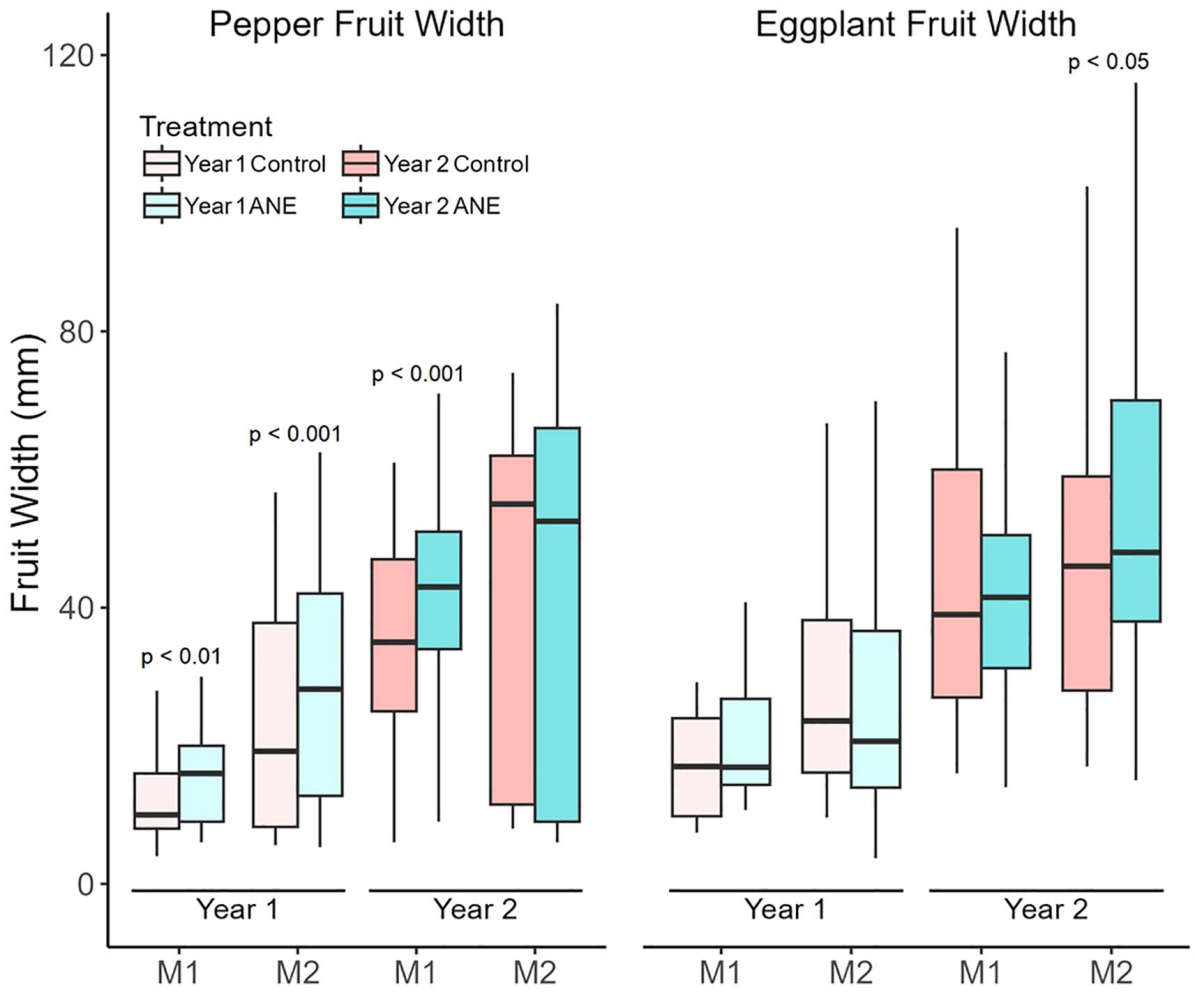
Width of developing pepper and eggplant fruits. Fruit width was measured twice during development in both years: once when the majority of first-set fruits were at the Cell division stage (M1), and again when they had reached the Fully expanded cell stage (M2). Data are presented as boxplots showing the median and interquartile ranges. In the first year, median fruit width increased significantly in ANE-treated pepper at both stages, but not in eggplant. In the second year, overall fruit width was larger in both species, but ANE treatment led to increased fruit width only in M1 peppers and M2 eggplants. Statistical significance was assessed using the Mann–Whitney U test.

Fruit size is another component, greatly influencing the yield. The fruit size (width) distribution of control- and ANE-treated plants is shown on **Figure 4**. Generally, pepper manifested substantial increase of its average fruit size for almost all measurements, while eggplant had a significant increase in only one instance (**Figure 4**).

### 2.2 ANE-induced metabolomic changes in pepper and eggplant

Metabolomic profiling was performed on leaves and fruits of both species over the two-year study. Fruits, sampled at the M1 and M2 time points were correspondingly in CD and FE developmental stages, while leaf samples were collected from the 6th or 7th terminal leaves when fruits were collected. These developmental stages were chosen, as they represent good matches for short- and long-term effects of ANE and they are important for seeing the immediate influence of the treatment over the young fruits, as well as the long-lasting changes on fully-grown fruits. Leaf samples are chosen as we are interested to reveal the source-sink relationship between leaves and fruits. The 6^th^/7^th^ terminal leaf is already a fully developed leaf and in the same time not yet senescent leaf, which makes it suitable for this purpose.

**Figure 5:**
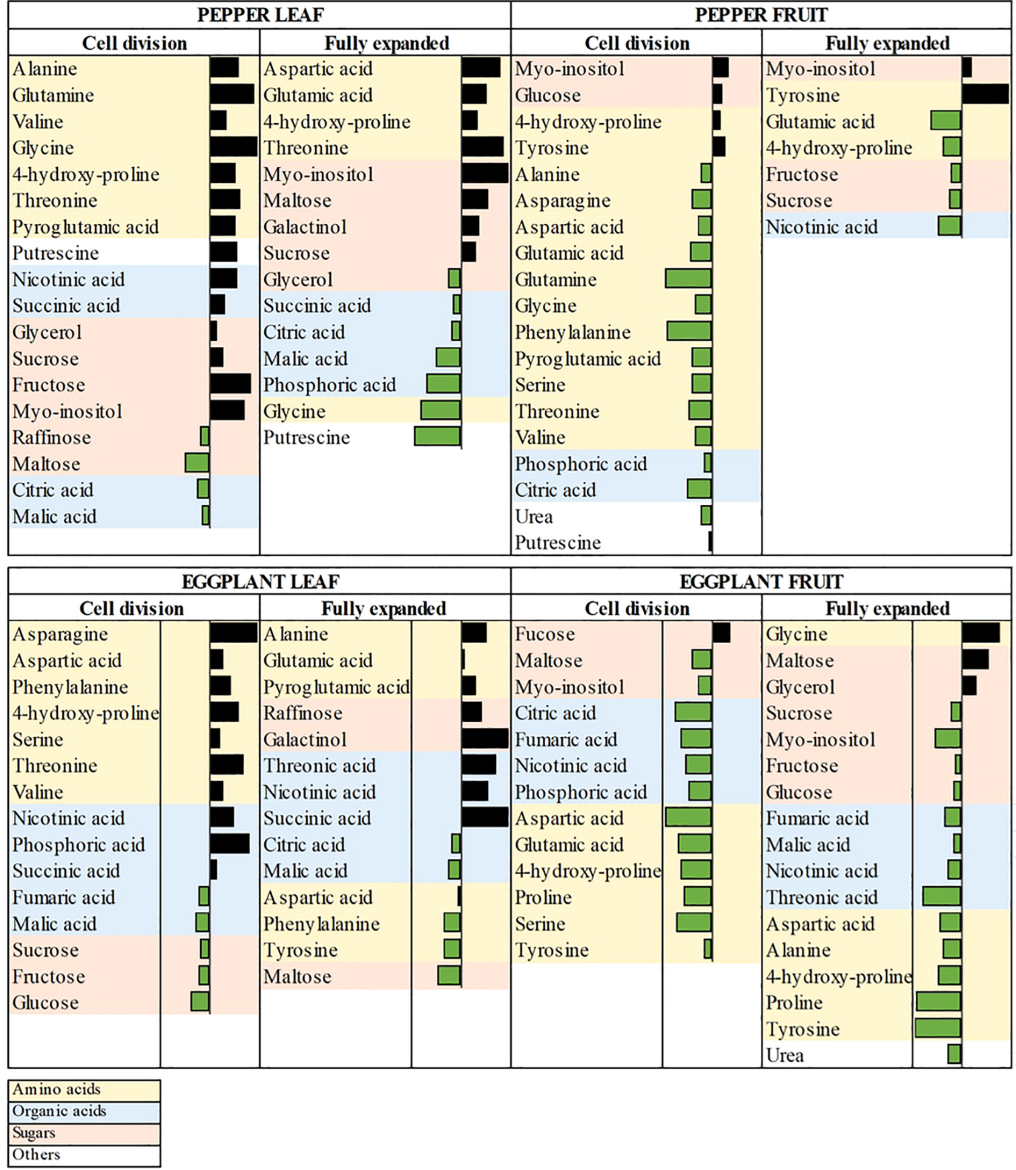
Pattern of thirty-two commonly differentially accumulated primary metabolite levels detected across two years field trial in ANE primed pepper and eggplant leaf and fruit samples. Data from the Cell division and Fully expanded stages of development are expressed as the average value of log2 fold change (ANE-treated vs. control samples) of metabolites from the two years in the form of data bars from maximum to minimum, which are distinguished by a threshold value (n=11).

Thirty-two of the all detected metabolites were common and shared between the years and the species. These of them, which manifested consistent pattern of increase/decrease in both years were considered key candidates for understanding the mechanisms, with which ANE-primed plants develop more fruits with improved yield, and their accumulation patterns are shown in **Figure 5**. The average fold-change values of these metabolites were visualized using data bar representations.

In primed pepper leaves, amino acids (e.g., alanine, 4-hydroxy-proline, threonine), sugars (e.g., sucrose, fructose) and sugar alcohols (glycerol, myo-inositol) exhibited an increasing trend. Notably, at the FE stage, organic acids showed a decline, unlike the CD stage, where some of them are elevated such as succinic acid and nicotinic acid. In primed pepper fruits, only a few metabolites, including sugars (e.g., glucose, maltose), sugar alcohols (myo-inositol) and amino acids (e.g., tyrosine, glycine, 4-hydroxy-proline), accumulated. Conversely, a large number of amino acids exhibited reduced accumulation at the CD stage, suggesting a shift in nitrogen metabolism favoring protein biosynthesis and fruit expansion.

In primed eggplant leaves, amino acid accumulation was observed predominantly at the CD stage, while sugars (e.g., sucrose, fructose, glucose and maltose) declined. Most organic acids, except for fumaric, malic, and citric acids, showed increased accumulation, indicating potential shifts in energy metabolism. In primed eggplant fruits, a few metabolites, such as fucose, maltose, glycine and glycerol, accumulated, while organic acids and most amino acids decreased, suggesting an adjustment in carbon flux toward growth-related processes.

Together the results show that ANE priming induced a metabolic shift favoring enhanced carbon allocation, nitrogen assimilation, and energy metabolism, particularly in leaves and early-stage fruits. Notably, these metabolic alterations likely contribute to improved fruit yield by optimizing carbon-nitrogen balance and enhancing energy efficiency.

### 2.3 Micro- and macronutrient composition is affected by ANE-treatment

To investigate the potential influence of the studied ANE extract on the micro- and macronutrient assimilation and distribution in the treated plants, quantification of various metal and metalloid chemical elements was carried out by ICP-MS. Tested samples included ANE-treated and untreated leaves and fruits of both crop species in the span of the 2 years. Raw data was obtained for a large population of elements, of which those for B, Ba, Ca, Co, Cr, Cu, Fe, K, Mg, Mn, Sr and Zn were processed and Ca, Cu, Fe, K, Mg, Mn and Zn were analyzed in more detail, due to their importance for plant development as well as human nutrition.

Overall, in most instances, the influence of the ANE extract was towards a slight reduction of the element quantity. This was most probably due to a nutrient dilution effect (Marles, 2017; Bhardwaj et al., 2024) caused by the augmented yield in both crops (**Figure 1**). Metal elements showing a reduction in content include: Ca, decreased in leaves and fruits of both crops; Fe, diminished in all samples except pepper fruits, in which only a non-significant tendency was observed; K, was depleted only in fully expanded pepper fruits and not considerably changed in the other samples; Mn, decreased only in eggplant while in pepper there was just a negative tendency; and finally – Zn, which was found to be diluted only in both of the tested developmental stages of pepper fruits, but not in pepper leaves and eggplants (**Supplementary Graphs S2**). Cu levels were also quantified, but the quality of the data was insufficient to draw conclusions.

**Figure 6:**
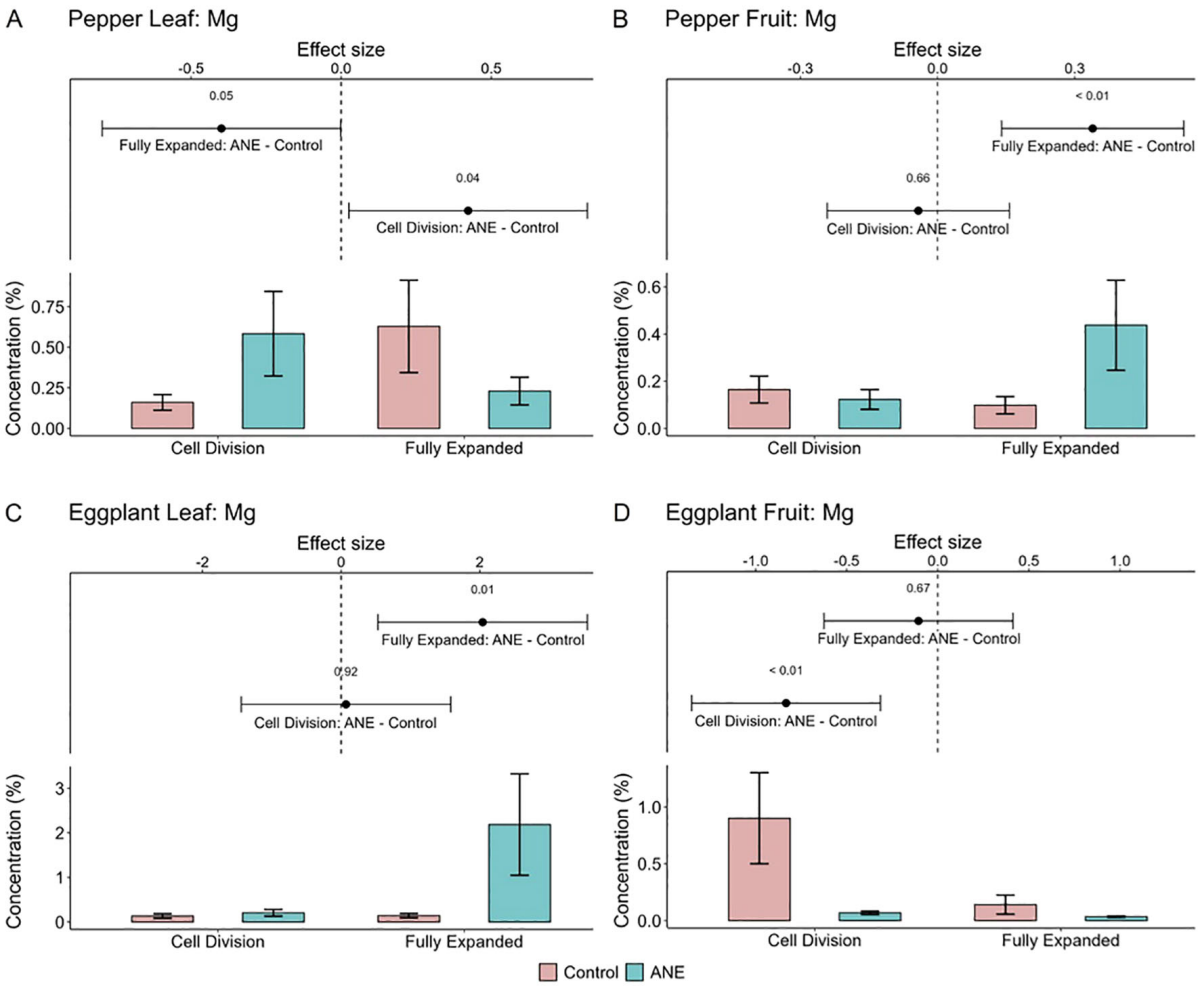
Effect of the ANE treatment on the concentration of Mg in pepper leaves **(A)** and fruits **(B)**, as well as eggplant leaves **(C)** and fruits **(D)** in two different stages of development – Cell division and Fully expanded cells. The data include 6 data points (triplicates from 2 consecutive years) for both crops. On the graph part the means and standard error for each of the samples are plotted. The effect size shows the direction of the ANE influence (negative or positive) and the 95% confidence intervals.

Interestingly however, the opposite outcome was observed for Mg, which increased in the fully expanded stage of the fruits of pepper, despite the nutrient dilution effect (**Figures 6A, B**). This was coupled with a concomitant reduction of Mg levels in the mature leaves, suggesting ANE-induced reallocation of Mg ions from the source to the sink organs. Notably, such a positive impact of the ANE extract on fruit Mg levels was recently reported in strawberries and raspberries as well (Kazakov et al., 2024), pointing to a possible trend observable in multiple crop species. On the other hand, in eggplants, after ANE-application a spike of Mg accumulation was detected only in the fully expanded leaves, but not in the fruits (**Figures 6C, D**).

It must be taken into consideration that the different cultivation locations (and thus differences in soil, temperature, weather and climate in consecutive years, see Materials and Methods and **Supplementary File S5**) resulted in high variation of the concentration of the studied elements, and could have influenced nutrient assimilation ability of the plants (**Figure 6**; **Supplementary Graphs S2**).

### 2.4 Transcriptomic analyses reveal genes, significantly impacted by priming with ANE

**Figure 7:**
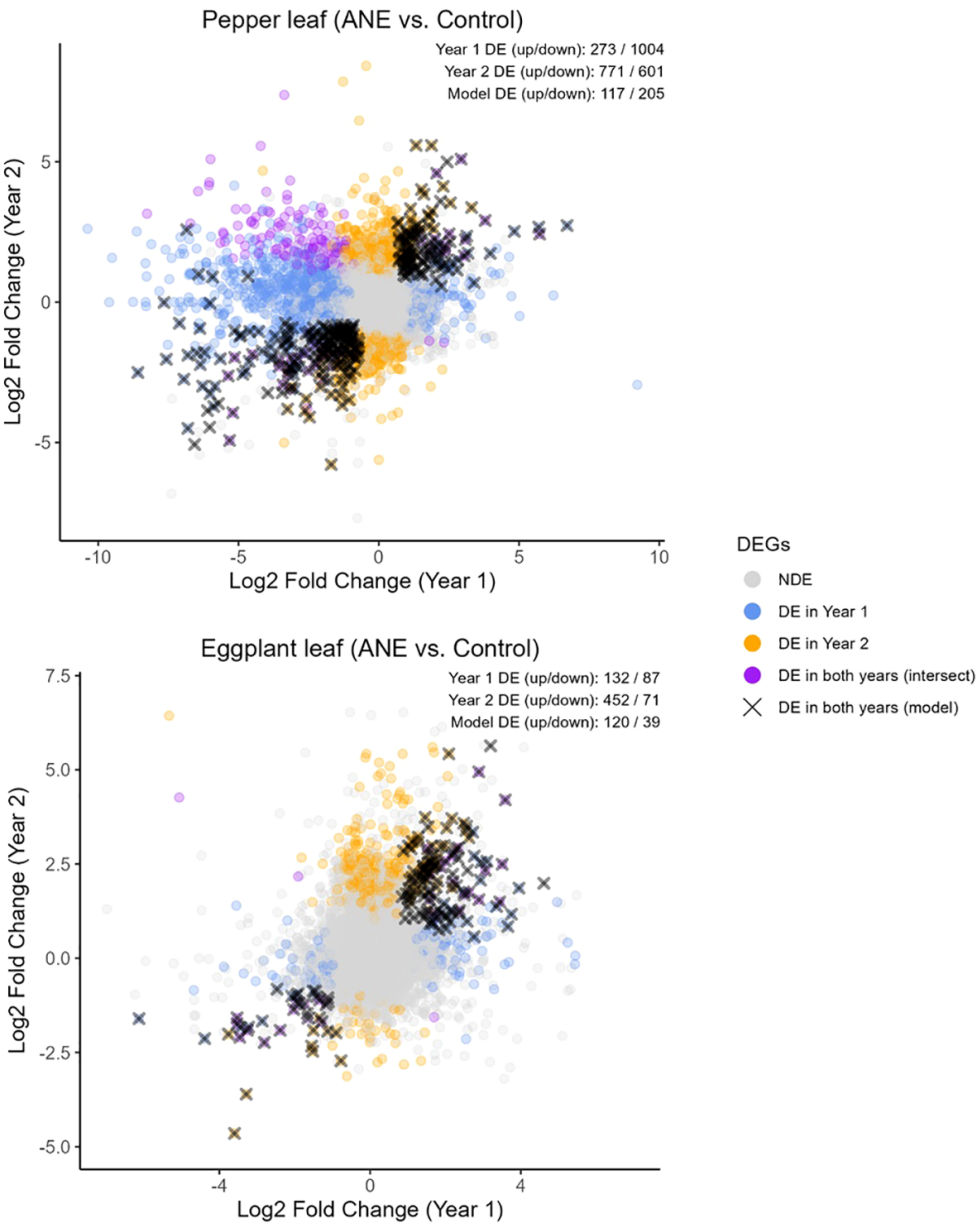
Differentially expressed genes in pepper and eggplant leaves. Changes in gene regulation were measured during early fruit development, when the majority of first-set fruits were at the cell division (CD) stage (5–8 days after ANE treatment). The plot shows log2 fold change (LFC) values (ANE-treated vs. control samples) for each gene in year 1 (x-axis) and year 2 (y-axis). Genes found to be significantly dysregulated in leaves in either year are highlighted in blue and orange, respectively, with overlapping DEGs in both years shown in purple. Genes that were assessed as consistently differentially regulated in the same direction across both years, based on a statistical model that accounted for year and treatment effects, are marked with a black cross and were considered the DEGs of interest in this study. The number of up- and down-regulated genes is indicated in the top right. Genes were considered differentially regulated if the adjusted p-value was below 0.05 and the absolute LFC exceeded 1.

Transcriptomic changes were measured at the CD stage (M1 time point) in both years to assess gene regulatory changes in adaptation to direct application of ANE, which could lead to downstream priming responses. RNA was extracted from both leaves and developing fruits of sprayed and control plants. Principal component analysis of the variance-stabilized data (VST-transformed) expression data separated the data primarily by year of the experiment and secondarily by control vs. treatment in both species (**Supplementary Figure S3**). Genes that were significantly impacted by ANE treatment similarly in both years (FDR < 0.05, absolute log^2^FC > 1) are highlighted in black in **Figure 7**. ANE treatment primarily influenced the gene regulation in leaves of treated plants, resulting in the modulation of many genes in pepper in both years (117 up, 205 down) and a smaller number in eggplant (120 up, 39 down). Low numbers of differentially expressed (DE) genes were observed in developing fruits at this stage, however (2 in pepper and 11 in eggplant). Shared DE genes were analyzed at the orthogroup level to identify gene families that were modulated similarly between both pepper and eggplant. Cross-species orthogroups were first identified using OrthoFinder3 and orthogroups that contained DE gene(s) from both species were identified. Only eight gene families were found to contain DE genes from both eggplant and pepper across both years (**Table 1**).

**Table 1:** Gene families modulated by ANE in both pepper and eggplant. The most closely-related A. thaliana orthologue for each gene is included.

Up-regulated Orthogroups	Down-regulated Orthogroups
β-1,3-glucanase (AT3G07320)	cysteine-type endopeptidase (AT1G62710)
putative membrane lipoprotein (AT4G29030)	putative cytochrome P450 monooxygenase (AT3G14660)
subtilisin-like serine endopeptidase (AT5G67090)	putative cytochrome P450 monooxygenase (AT3G48270)
heavy metal transport/detoxification superfamily (AT1G23000)	
cinnamate-4-hydroxylase (AT2G30490)	

The up-regulated DE gene sets in leaves in the two species were enriched for terms related to carbohydrate metabolism and the cell wall (cellulose, pectin, xylan, lignin metabolism), while the down-regulated DEGs shared a few terms related to oxidative stress and iron homeostasis. However, only the single term GO:0030244 (cellulose biosynthetic process) was specifically shared across both species, in the up-regulated gene sets (**Supplementary Table S4**).

## 3 Discussion

### 3.1 Improving the yield and its components by ANE-treatment

Previous data report that biostimulants improve yield under both normal and stress conditions (Yakhin et al., 2017). In particular, seaweed extracts exert a positive impact on yield and harvest quality on a variety of crops, such as wheat, rice, tomato, cucumber, broccoli, spinach, bean, etc (Bulgari et al., 2019; VanOosten et al., 2017). Additionally, it has been shown that the same *Ascophyllum nodosum* biostimulant used in this study enhances yield during salt stress in tomato (DiStasio et al., 2020) and stimulates the growth of roots and shoots in tomato seedlings (Popescu, 2016). Furthermore, recent data shows that this ANE extract enhances yield in greenhouse-grown raspberry and strawberry (Kazakov et al., 2024) and in tomato subjected to drought stress (Kanojia et al, 2024). This prompted us to investigate the influence of the ANE on the growth and yield of other important crops under normal field conditions.

The studies were done on two economically important vegetable crops – pepper and eggplant. For these vegetables, even a slight improvement in the yield or ripening times could have a very significant agricultural impact, as earlier harvesting times result in fruits being placed into higher pricing categories. This study adds to the existing data on the effects of ANE use on crop yield, specifically under open-field conditions. Two separate agricultural fields were used for plant cultivation, aiming encompassing more variable agricultural conditions in the two consecutive years (see Methods 4.1), and treatments were reproduced as exactly as possible. The main variations were in the climatic conditions during the different growth seasons (See **Supplementary File S5**) and local soil differences, and corresponding to them variable speeds of plant development. The two distinct agricultural crops were examined simultaneously also as a means of comparison of any potential effect of ANE over different plant species.

In both crops, ANE treatment led to a significant increase in the yield (**Figures 1A**, **2A**), especially at the first harvest (**Figure 2D**). The second harvest was lower than the control groups, which indicates that the pool of the first fruit set was exhausted, as a higher number of fruits grew and matured faster, leading to a greater proportion being picked during the first harvest, leaving fewer fruits available for the second harvest. Overall, the combination of both harvests was still higher for ANE-treated variants (**Supplementary Table S1**). The higher yield was primarily due to the higher number of fruits that treated plants bear in comparison to controls, which was more pronounced in eggplant than pepper (**Figures 1B**, **2B**). During the first year, only ANE-treated pepper showed a significantly higher average fruit weight (**Figure 1C**), an effect that had the same observed trend in both crops in the second year (**Figure 2C**).

Additional data were gathered and analyzed, concerning the fruit number and fruit size (width) distribution, during two developmental stages preceding harvest. These elucidated the pattern of development, in its temporal aspect, influenced by ANE treatment. During the first year, the fruit number increased significantly in all ANE-treated groups, in both plant species, for both pre-harvest stages (**Figure 3**), which is in concordance with the same observation at harvest (**Figure 2B**). For the second year though, this increase was still observable, but it was much weaker at the pre-harvest stages, lacking sufficient significance. Nevertheless, at the time of harvest, fruit number was still in favor of the ANE-treated group (**Figure 2B**), likely due to the faster maturation and earlier picking of more numerous fruits, supporting the suggestion that ANE speeds up fruit development and maturation.

Why ANE is causing increase in the fruit number, is a matter, deserving further investigation, although two main reasons might apply: 1. ANE causes an increase in the number of flowers, or 2. ANE enhances the process of formation and/or retention of fruit sets by influencing some of its required components, such as pollination/fertilization efficiency, fruit set abortion, early fruit set losses and further fruit survivability at later stages. Having in mind that ANE was applied after most of the flowering buds from the first set were formed and at the time of early flowering, the latter reason is more probable, especially when many more possible sub-processes are involved and could be influenced. It must be noted though, that a third possible reason for increasing the fruit number may apply: the faster maturation and ripening of the harvestable fruits from ANE-treated plants (see the previous paragraph and **Supplementary Table S1**).

Regarding fruit size (width) – a stable trend of increasing upon ANE treatment was observed in pepper during almost all measurements, which wasn’t the case for eggplant, where only one measurement clearly shows slightly increased average width (**Figure 4**). This most probably suggests different sensitivity over this parameter of the two distinct plant species.

### 3.2 Metabolic priming and its role in increased fruit yield

Metabolomic profiling of pepper and eggplant leaves and fruits from the two consecutive growing seasons revealed significant metabolic changes in response to foliar application of the biostimulant. The metabolomic changes shown in **Figure 5** indicate improved carbon allocation, nitrogen assimilation, and osmoprotection, all of which are crucial for plant growth and productivity under field conditions. These findings align with previous studies demonstrating that metabolic priming via biostimulants can enhance source-sink relationships, facilitating improved nutrient translocation and fruit development (du Jardin, 2015; Kanojia et al, 2024).

Nitrogen metabolism is a crucial determinant of fruit yield and quality, as amino acids serve as precursors for both structural proteins and secondary metabolites (Martínez-Rivas and Fernie, 2024). One of the key metabolic reprograming effects, observed in primed leaves was the increased accumulation of amino acids. This trend might point to enhanced nitrogen assimilation and protein biosynthesis, essential for sustained cell division and fruit enlargement (Pratelli and Pilot, 2014). Additionally, the observed decrease in amino acid in primed fruits further suggests that these amino acids are rapidly incorporated into protein synthesis rather than accumulating in free form, optimizing nitrogen use efficiency (Hildebrandt et al., 2015). This suggests a possible metabolic adaptation in ANE-primed plants that enhances source-to-sink allocation, allowing for sustained fruit growth and improved nutrient mobilization (Tegeder and Masclaux-Daubresse, 2018).

A notable increase in sugars and sugar alcohols, particularly in primed pepper leaves, such as myo-inositol, fructose, sucrose and galactinol, was observed. These sugars are essential for energy storage, carbon partitioning, and osmotic regulation, all of which contribute to fruit biomass accumulation and expansion (Smeekens et al., 2010; Ruan, 2014; Loewus and Murthy, 2000; Kanayama, 2017; Du et al., 2024). Sucrose is a key photoassimilate transported from leaves to fruits, directly influencing fruit biomass accumulation (Patrick et al., 2013). In turn, myo-inositol, plays a pivotal role in cell wall biosynthesis and phloem loading, facilitating efficient carbon transport towards developing fruits (Loewus and Murthy, 2000). The accumulation of sugar alcohols like galactinol and myo-inositol also supports the hypothesis of enhanced stress tolerance and osmoprotection in primed plants (Taji et al., 2002). Complementing these metabolomic findings, the transcriptomic analysis revealed that up-regulated DEGs in primed leaves were significantly enriched for GO terms associated with carbohydrate metabolic processes, supporting the observed enhancement in carbohydrate metabolism.

Previous studies have shown that under conditions favoring enhanced growth, plants tend to shift from energy-intensive respiration towards biosynthetic pathways that support fruit expansion (Carrari et al., 2006; Sweetlove et al., 2010). The reduction of malic acid, citric acid, and fumaric acid in both leaves and fruits suggests a downregulation of the tricarboxylic acid (TCA) cycle, possibly optimizing respiration efficiency to favor growth over excess energy dissipation. Additionally, reduced malate and citrate levels might indicate a higher flux of intermediates toward biosynthesis of amino acids and secondary metabolites rather than oxidative metabolism (Maurino and Engqvist, 2015).

The polyamine putrescine, involved in stress tolerance and cell proliferation, has been linked to enhanced fruit set under both normal and suboptimal conditions (Gill and Tuteja, 2010; González-Hernández et al., 2022). Also, proline derivatives such as hydroxyproline act as osmoprotectants, stabilizing proteins and membranes under stress conditions (Kavi Kishor and Sreenivasulu, 2014). The accumulation of 4-hydroxy-proline and putrescine in primed plants at Cell division stage suggests enhanced osmotic adjustment and stress tolerance (Szabados and Savouré, 2010; Gill and Tuteja, 2010). Interestingly, lower levels of proline and 4-hydroxy-proline in eggplant primed fruits might indicate an altered stress-response mechanism, where plants rely on metabolic efficiency rather than stress-induced accumulation of protective metabolites. This finding aligns with studies suggesting that plants with optimized growth often prioritize biosynthetic and developmental pathways over stress-related metabolite accumulation (Szabados and Savouré, 2010; Sweetlove et al., 2010). Consistent with these findings, transcriptomic analyses revealed that down-regulated DEGs in primed tissues were enriched for GO terms related to oxidative stress responses, indicating a potentially reduced oxidative burden and enhanced antioxidant regulation in primed plants.

The observed metabolomic shifts in ANE-treated leaves suggest an adjusted physiological state that directly stimulates fruit growth and yield. Increased sugar transport, nitrogen assimilation, energy metabolism, and osmoprotection collectively create a favourable metabolic environment that supports the increased fruit yield. These findings highlight the potential of biostimulant applications in modulating plant metabolism to achieve higher productivity and sustainable agriculture.

### 3.3 Effects of ANE-priming on nutrient abundance

The ICP-MS quantification of metals and metalloids in this study was carried out with the aim to elucidate whether the treatment with the biostimulant exerts an effect on the micro- and macronutrient element composition. This could have a significant impact both for the crops themselves, especially the micronutrient concentration in the leaves, and for humans, mainly in the mature fruits, when the latter are consumed as food. In plants, these metal elements are involved in virtually all aspects of physiology and development, from primary metabolism to reproduction (Hänsch and Mendel, 2009). In humans, their role is not less important, as they are crucial for preserving good health and preventing some diseases (Höller et al., 2018). Therefore, the metal composition of the fruits is an important marker of food quality. In both types of organisms, metal elements are mostly incorporated in the structure of a large number of enzymes and other proteins, participating in signal transduction pathways as well as genetic control mechanisms, and contributing to the redox balance (Fraga, 2005).

As discussed in the results section above, in most of the cases, the quantity of the measured elements was less in the ANE treated samples. This is hardly surprising, considering the observed increase in the fruit yield of both pepper and eggplant. Such a negative correlation between the yield and the amount of nutrients is usually designated as nutrient dilution effect, and this has been reported in previous occasions for various crop species (Morris and Sands, 2006; Davis, 2009), probably caused by a combination of factors like preferential accumulation of primary metabolites like sugars and organic acids in rapidly growing organs, depletion of the trace elements in the soil or limited assimilation capacity of the crops themselves. Nevertheless, the results for Mg in pepper fruits demonstrated a clear opposite trend for higher accumulation after ANE treatment. This is consistent with another study with the same biostimulant, but in raspberry and strawberry, confirming that this *Ascophyllum nodosum* product can lead to Mg biofortification of certain fruits. This finding is important in the light of human nutrition, considering the large impact of Mg and its deficiency on health and wellbeing.

Mg is the fourth most abundant metal element in humans and can constitute up to 20–28 g of the body mass (Fiorentini et al., 2021). In over 600 enzymatic reactions Mg is used as a cofactor. At the organism level it is vital for proper bone development, muscle contraction, blood pressure regulation and neuromuscular function, etc., while in the cells it participates in signaling pathways, transmembrane transport, energy storage and transfer, carbohydrate, lipid and protein metabolism, DNA and RNA stability, and cell proliferation (Al Alawi et al., 2018; Fiorentini et al., 2021). However, a significant proportion of the population do not consume the recommended Mg daily doses, making hypomagnesaemia a common problem (de Baaij et al., 2015). The risks associated with this condition arise from the fact that in general milder cases do not lead to clinically significant symptoms until serum levels drop below 0.5 mmol/L, when complications such as neuromuscular hyperexcitability and neuropsychiatric disturbances can manifest (Pham et al., 2014). Therefore, increasing the Mg concentration in common food sources, including by stimulation with ecologically friendly bioactives like ANE extracts, can contribute to counteracting this widespread nutrient deficiency.

### 3.4 Insights into gene regulation, caused by ANE-priming

These results add to the growing literature, investigating the gene regulatory adjustments to biostimulant treatment in crops. While the transcriptomic reprograming varied strongly by year of treatment (**Supplementary Figure S3**), a subset of genes were modified similarly across both experimental years (**Figure 7**). Dysregulated genes included those associated with growth & development (particularly cellulose and carbohydrate transport) and stress, as evidenced by the enrichment of associated GO terms in the pepper and eggplant gene sets independently. However, only a very small number of orthologous gene families were differentially expressed across both species and so might be related to a speculated common answer to ANE treatment (**Table 1**). Putative roles for these gene families in modulating biostimulant adaptation are hard to ascertain as they play diverse roles in metabolism, signaling and stress response. β-1,3-glucanase is a pathogensis-related (PR) protein primarily involved in biotic stress response but is also involved in development (Benitez-Alfonso et al., 2013; Fang et al., 2024). Similarly, subtilisin-like proteases play broad roles in plant defense and signaling (Figureiredo et al., 2014). Heavy metal transport/detoxification proteins and cinnamate-4-hydroxylases are involved in cellular homeostasis of heavy metals and biosynthesis of phenylpropanoids, respectively, both processes broadly implicated in cellular metabolism at multiple levels (Hall, 2002; Vogt, 2010). A deeper investigation into the long-term effects of ANE treatment on gene regulation and plant development may be necessary to discover what pathways result in altered fruit development days to weeks after initial priming.

## 4 Materials and methods

### 4.1 Plants, cultivation conditions and treatments

Plant material – two crop species – pepper (*C. annuum* var. Amareta F1) and eggplant (*S. melongena* var. Black Pearl F1) were included in the trial. The seeds were germinated directly on soil in small plug trays and seedlings were raised in greenhouse until reaching four- to six-leaf stage. Then plants were transferred to the field, directly in the soil and grown under open-air conditions, surrounded by plastic mulch and with drip fertigation. The treatments were reproduced as exactly as possible in two separate replications across the two growth seasons. For ease of operation and simulation of real agricultural conditions, whole rows were planted next to each-other and treated differently either as Control or as ANE-primed variants.

Location – the plants were cultivated at two different plots of land, at different geographic locations and belonging to separate organizations: OporaZaden’s trial field, near Tsalapitsa village (42°11’49”N 24°34’07”E) and Maritsa Vegetable Crops Research Institute’s (MVCRI) trial field, near Plovdiv (42°10’30”N 24°45’49”E), positioned approximately 20 km afar. The hosting organizations took care for seedling production, soil cultivation, planting, irrigation/fertigation and pests & diseases management.

ANE – the extract, used in this study is commercially available SuperFifty Prime^®^, obtained from BioAtlantis Ltd., (Tralee, Ireland). The storage, handling, dilution and all other work with this substance was done, according to manufacturer’s recommendations. SFP can be stored for up to 3 years at temperatures above 5 °C, avoiding direct sunlight. It is recommended to avoid spraying with SFP in case of rainfall. If it rains immediately after SFP foliar application and before it dries up, the treatment should be repeated at convenient time, but not before all the rain water evaporates completely. SFP is compatible with most other agricultural chemicals for pest management and fertilizers. The manufacturer recommends before mixing in the main tank, small volumes to be test-mixed in separate vessel to determine compatibility and SFP to be added last to the mixture.

Treatment – all plants were treated with 0.4% ANE, using an electrical or a hand back sprayer with fine droplet size. ANE working solution was prepared with water from the local sources (filtered groundwater), used for the irrigation and fertigation systems. Control groups were treated with water only. Plants were sprayed from all sides and care was taken to ensure spraying of the flowers. To avoid the formation of larger droplets, the jet flow was adjusted to fine mist. Special attention was given to ensure that sprayings were not performed during the hottest time of the day, when sunshine is at its full intensity, to prevent sunburns.

Pepper: The first ANE foliar application was performed when most plants had either one flower bud, soon to open, or were entering the early blooming phase. The second foliar application was executed after 15 and 11 days (1st and 2nd year respectively) of the first spray at the full bloom stage.

Eggplant: The first ANE spraying was performed when most plants had their first flower opened, and the second ANE treatment was performed at the full bloom stage after 10–11 days of the first spray.

Fruit size parameters - to monitor fruit development progression in treated and untreated plants, fruit diameter was measured, using calipers, at two time points – Measurement 1, called also Cell Division (CD) stage of the first fruit set, as the fruits are still very small and actively growing (corresponding to Stage 712 from BBCH Scale: First fruitlet abscission - ovaries are green, the diameter of retained fruits is 5–10 mm) and Measurement 2, called also Fully Expanded cell (FE) stage of the first fruit set, as the fruits almost reached their maximum size (corresponding to Stage 718–719 from BBCH Scale: when the fruits reach 80%-90% or more of their final fruit size). At each measurement time point, the total number of fruits were counted and after harvest, the yield was weighed.

### 4.2 Sampling for omics

From treated and untreated eggplant and pepper plants, 6th or 7th terminal leaves and fruits at Cell division, and Fully expanded cell phases were harvested for multi-omics studies. The samples were flash-frozen in liquid nitrogen and stored at -86°C. The frozen leaf material and the fruits sampled at the Cell division phase were collected and kept in 50 ml falcon tubes, fruits from Fully expanded cells were wrapped separately in aluminum foil and stored in groups. Total leaf samples were roughly crushed in-tube in moderately sized pieces before proceeding with the milling on powder. The total amount of the small fruit samples from the Cell division phase were roughly crushed in medium pieces with a pre-chilled steel hammer on a plastic kitchen board and transferred for milling. The fruits from Fully expanded cells phase were pre-crushed with a pre-chilled steel hammer on a plastic kitchen board and equal amount of pieces from all main parts of the fruit (with a volume of about 40 ml) were transferred for milling. The milling to fine powder was done on a ball mill VWR^®^, Beater Mixer Mill in 50 ml pre-chilled stainless still grinding jars, with a single, 14 mm stainless still grinding ball at vibrational frequency 30 Hz for one minute. If needed, the treatment was repeated.

### 4.3 Metabolite profiling

~50 mg frozen powdered plant material was homogenized in 700 μL of ice-cold 100% methanol at RT for 15 min and 350 μL of chloroform followed by addition of 700 μL water. 150 μL of the polar fraction was dried under vacuum and used for analysis. The remaining amounts were stored at -20°C.

Analysis by gas chromatography coupled with mass spectrometry was performed using the same equipment set up and protocol as described (Lisec et al., 2006). Briefly, the dried residue from the extraction was derivatized for 120 min at 37°C (in 40 μL of 20 mg/mL - 1 methoxyamine hydrochloride in pyridine) followed by a 30 min treatment at 37°C with 70 μL of MSTFA. An auto-sampler Gerstel Multi-Purpose system (Gerstel GmbH &Co.KG) was used to inject the samples to a chromatograph coupled to a time-of-flight mass spectrometer (GC-MS) system (Leco Pegasus HT TOF-MS (LECO Corporation)). Helium was used as a carrier gas at a constant flow rate of 2 mL/s and gas chromatography was performed on a 30 m DB-35 column. The injection temperature was 230°C and the transfer line and ion source were set to 250°C. The initial temperature of the oven (85°C) increased at a rate of 15°C/min up to a final temperature of 360°C. After a solvent delay of 180 s, mass spectra were recorded at 20 scans s-1 with m/z 70–600 scanning range. Chromatograms and mass spectra were evaluated by using Chroma TOF 4.5 (Leco) and TagFinder 4.2 software. Metabolites were annotated based on a retention index calculation with deviation <5% and compared with the reference data of the Golm Metabolome Database, http://gmd.mpimp-golm.mpg.de (Luedemann et al., 2008).

### 4.4 Determination of metals and metalloids by ICP-MS

Approximately 0.25 g of homogenized and freeze dried samples were weighed into PFA MARSXpress vessels (Mars 6, CEM Corporation, Matthews, NC, USA) and 0.5 mL trace metal grade concentrated HNO3 and 2 mL 30% H2O2 was added to each vessel.

The samples were left for 30 min before being placed in the microwave and digested in closed vessels according to Miller’s protocol (Miller, 1998). Duplicates of samples and method blanks were prepared and digested in a single batch and later diluted to 15 mL with reagent water.

The calibration standards and QC standards were prepared using an ICP Multi-element Standard Solution IV Certipur^®^ (Merck, Rahway, NJ, USA) and most of the elements were calibrated from 0.01 to 10 ppm. The calibration curves were based on seven standard solutions, including a blank.

The 7850 ICP-MS (Agilent Technologies, Santa Clara, CA, USA) was used for the measurement of all analytes. The system was fitted with Ultra High Matrix Introduction system and ORS4 cell operating in helium (He) mode, which reduces common polyatomic interference. The ICP-MS was fitted with glass concentric nebulizer, quartz spray chamber and torch with 2.5 mm id injector. Additionally, the operating conditions of the ICP-MS were as follows: RF power 1600 W, plasma argon flow rate 15.0 L min−1, nebulizer gas flow rate 0.9 L min−1.

### 4.5 RNA isolation, sequencing and transcriptomic analysis

Using deeply frozen powdered plant material, total RNA was isolated with Quick-RNA Miniprep Kit by Zymo Research, following the instructions of the manufacturer. The ratios of absorbance at 260/280 and 260/230 nm was used to assess the purity of the isolated RNA. The RNA integrity was determined by visualization on agarose gel.

Library preparation and RNA sequencing was performed at BGI, Hong Kong. Raw 150bp paired-end reads were quality checked using FASTQC (Andrews, 2010) before and after preprocessing using Trim Galore (https://www.bioinformatics.babraham.ac.uk/projects/trim_galore/). Processed reads were mapped against the pepper (PLAZA Dicots v5; Van Bel et al., 2022 and Kim et al., 2014) and eggplant (solgenomics.net; Barchi et al., 2021) genomes using Salmon selective alignment (Patro et al., 2017). The mapping rate against the transcriptome was lower in pepper than eggplant (55% vs. 75%) but consistent across all samples from each species. Differential expression analysis was performed using the Salmon/tximport/DESeq2 pipeline and the model design “~year+treatment” to account for variability across both years (Love et al., 2014; Soneson et al., 2015). Genes with an FDR < 0.05 and absolute log2 fold change (log2FC) > 1 were considered significantly altered in expression. GO enrichment analysis was performed using TopGO (Alexa and Rahnenfuhrer, 2024) using the weight01 algorithm. For orthologous gene analysis, shared orthogroups between pepper and eggplant were identified using OrthoFinder3 (Emms and Kelly, 2019) and the proteomes of several dicot and monocot species: *A. thaliana, A. lyrata, B. rapa, C. annuum, F.* x *anannassa, S. melongena, S. lycopersicum, R. idaeus, V. vinifera, Z. mays, O. sativa*, and *S. bicolor*. Data analysis and figures were done in R (Core Team, 2021), using the tidyverse package (Wickham et al., 2019).

### 4.6 Statistical and data analyses

Physiology: All data were tabulated in Microsoft Excel, where most of the statistical processing was done. Any measurements falling outside the 1.5 interquartile range of their respective group were discarded as outliers. Data were expressed as average with added standard error of the mean. Differences between two single treatments were tested by Student’s t-test, with p ≤ 0.05 considered statistically significant.

The statistical analyses and visualizations of **Figures 1**, **6** and **Supplementary Graphs S2** were realized using R 4.3.1. Mixed linear models were utilized with the help of the lme4 package (Bates et al., 2014). For **Figure 6** and **Supplementary Graphs S2**, treatment with ANE extract and cell stage were modeled as main effects, together with their interaction. The effect of year of growth on the measurements was modeled as a random effect. Estimated marginal means and effect sizes were calculated with the emmeans package (Lenth, 2024). The results were visualized with the help of ggplot2 (Wickham, 2016), ggpubr (Kassambara, 2023) and cowplot (Wilke, 2024). A model was fitted for each element and each plant tissue. The presented values are of the estimated marginal means, the standard errors of the means, the mean differences and the 95% confidence intervals of the mean differences (95% CI) of each individual model.

## 5 Conclusion

**Figure 8:**
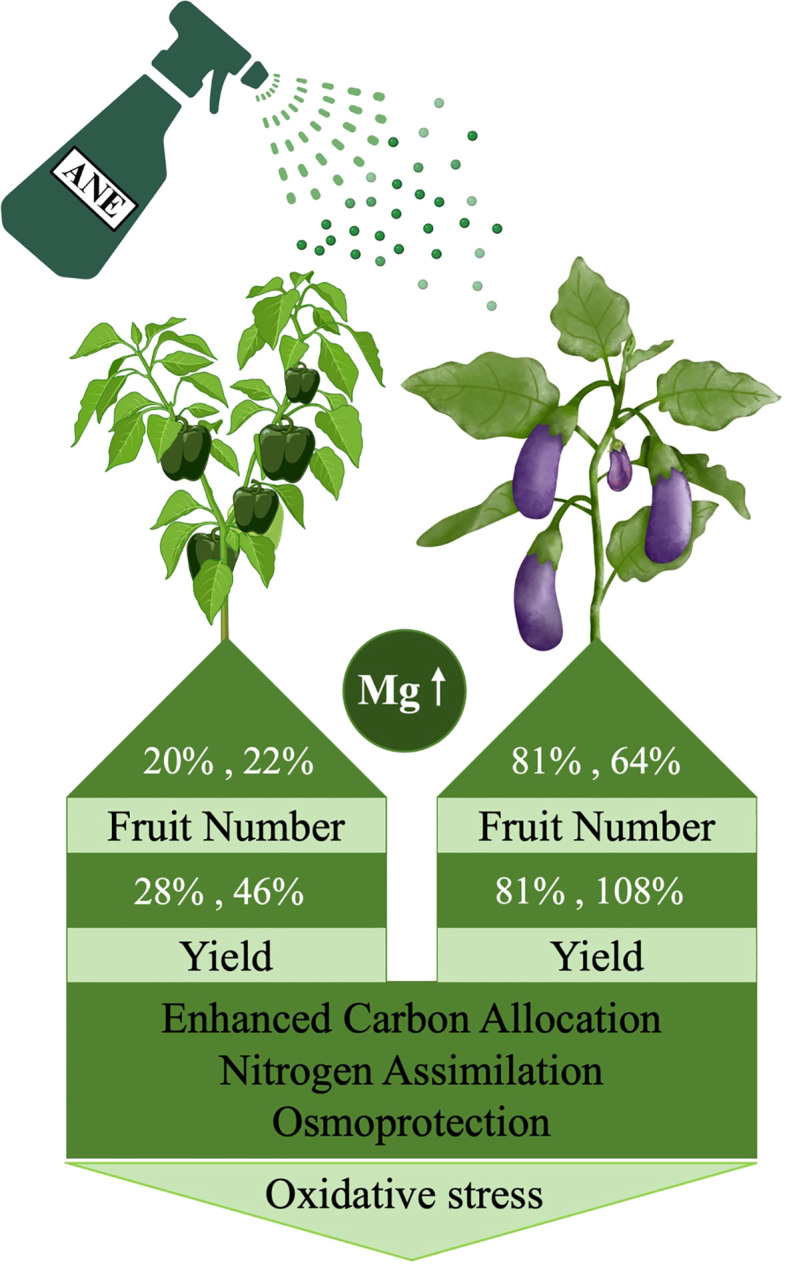
Illustrated figure, summarizing the effect of ANE treatment on yield improvement in pepper and eggplant via key metabolic changes, Mg accumulation, enhanced osmoprotection, and reduced oxidative stress, as evidenced by transcriptomic analysis. The figure was created using BioRender.com.

This study shows that foliar application of an *Ascophyllum nodosum* extract (ANE) significantly enhanced fruit yield in both pepper and eggplant under open-field conditions. As summarized in the model in **Figure 8**, yield improvement was primarily driven by an increased number of fruits, with species-specific effects on fruit size. Metabolomic profiling revealed that ANE priming induces distinct metabolic shifts in source and sink tissues, characterized by enhanced carbon allocation, nitrogen assimilation and osmoprotection, which likely contribute to improved fruit development and productivity. These metabolic changes were supported by transcriptomic data, which identified gene expression changes related to carbohydrate metabolism and oxidative stress regulation in treated leaves. Furthermore, the ANE treatment was associated with nutrient dilution effects in several elements, although a notable increase in magnesium levels was observed in pepper fruits, suggesting a potential for crop biofortification. Overall, the findings provide insights into how ANE modulates physiological and molecular reprograming to improve fruit yield and quality supporting its broader application in sustainable horticultural production.

## Data Availability

The datasets presented in this study can be found in online repositories. The names of the repository/repositories and accession number(s) can be found in the article/**Supplementary Material**.
