# Supplementary figures and images for: Sustainable agriculture through seaweed biostimulants: a two-year study demonstrates yield enhancement in pepper and eggplant
Source: Front Plant Sci. 2025 Sep 8;16:1655340. doi: 10.3389/fpls.2025.1655340 (PMC12450877; doi:10.3389/fpls.2025.1655340)

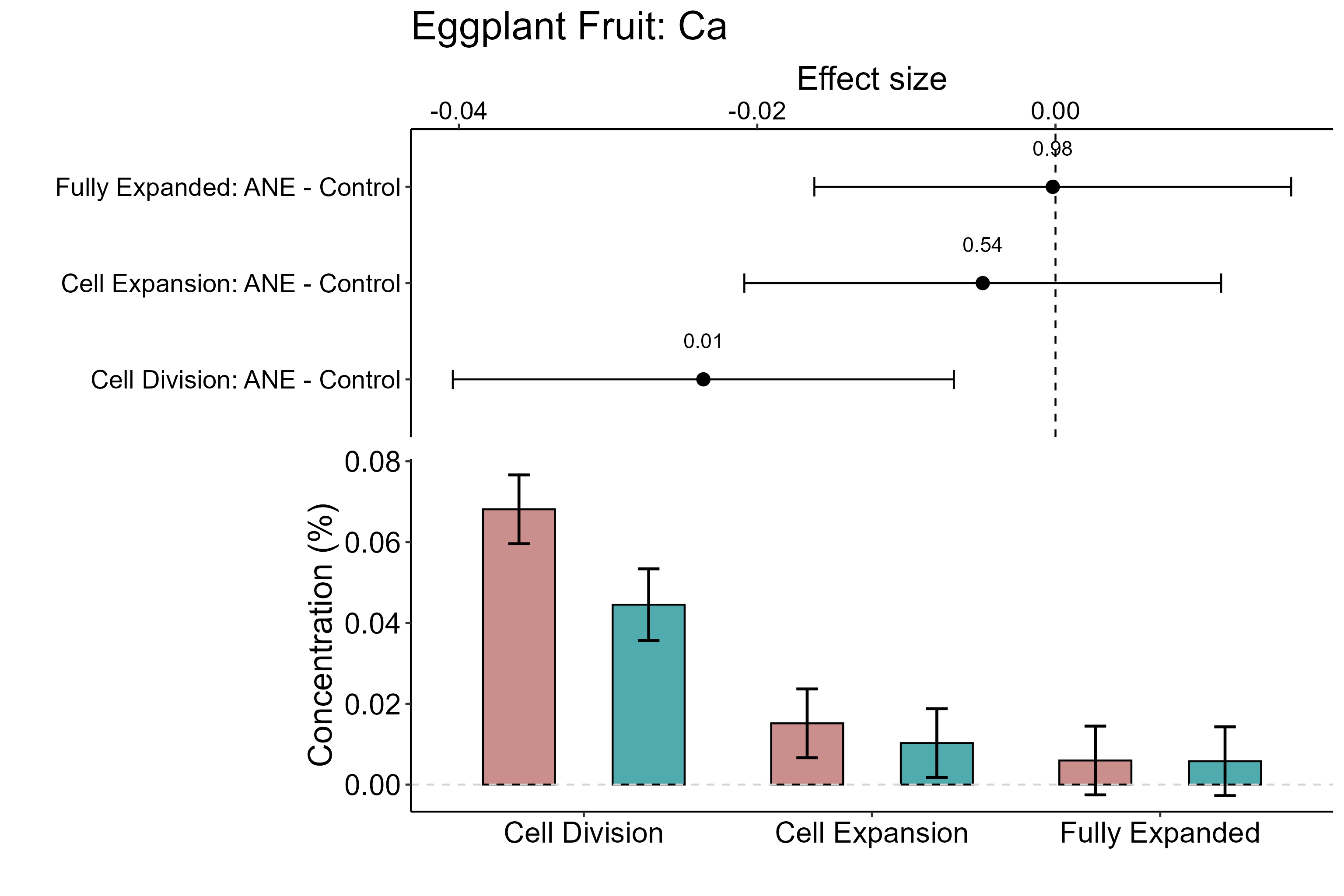

Supplement: Supplementary file 1 [file DataSheet1.zip › Micronutrients_barcharts/Eggplant_Fruit_Ca.png]

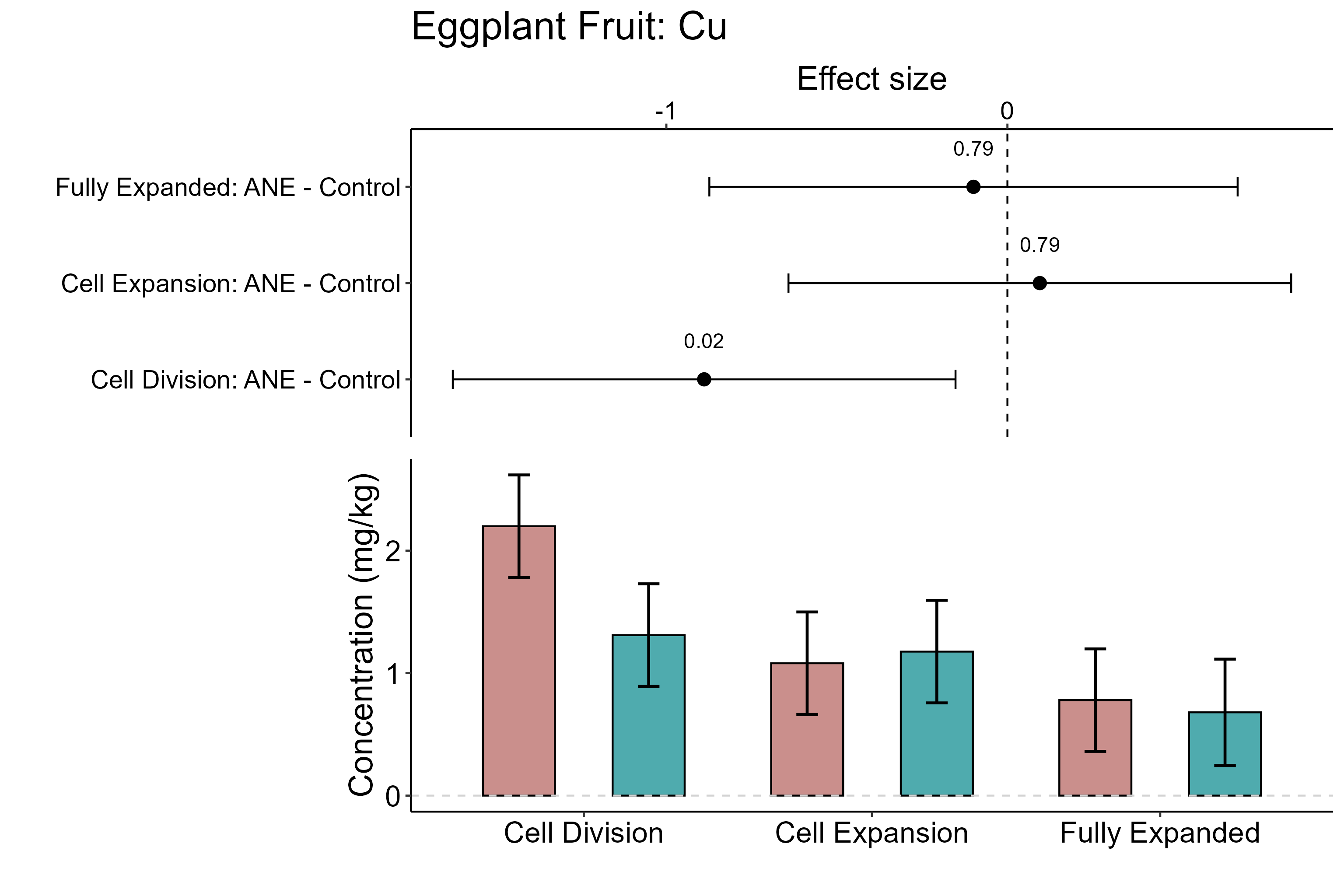

Supplement: Supplementary file 1 [file DataSheet1.zip › Micronutrients_barcharts/Eggplant_Fruit_Cu.png]

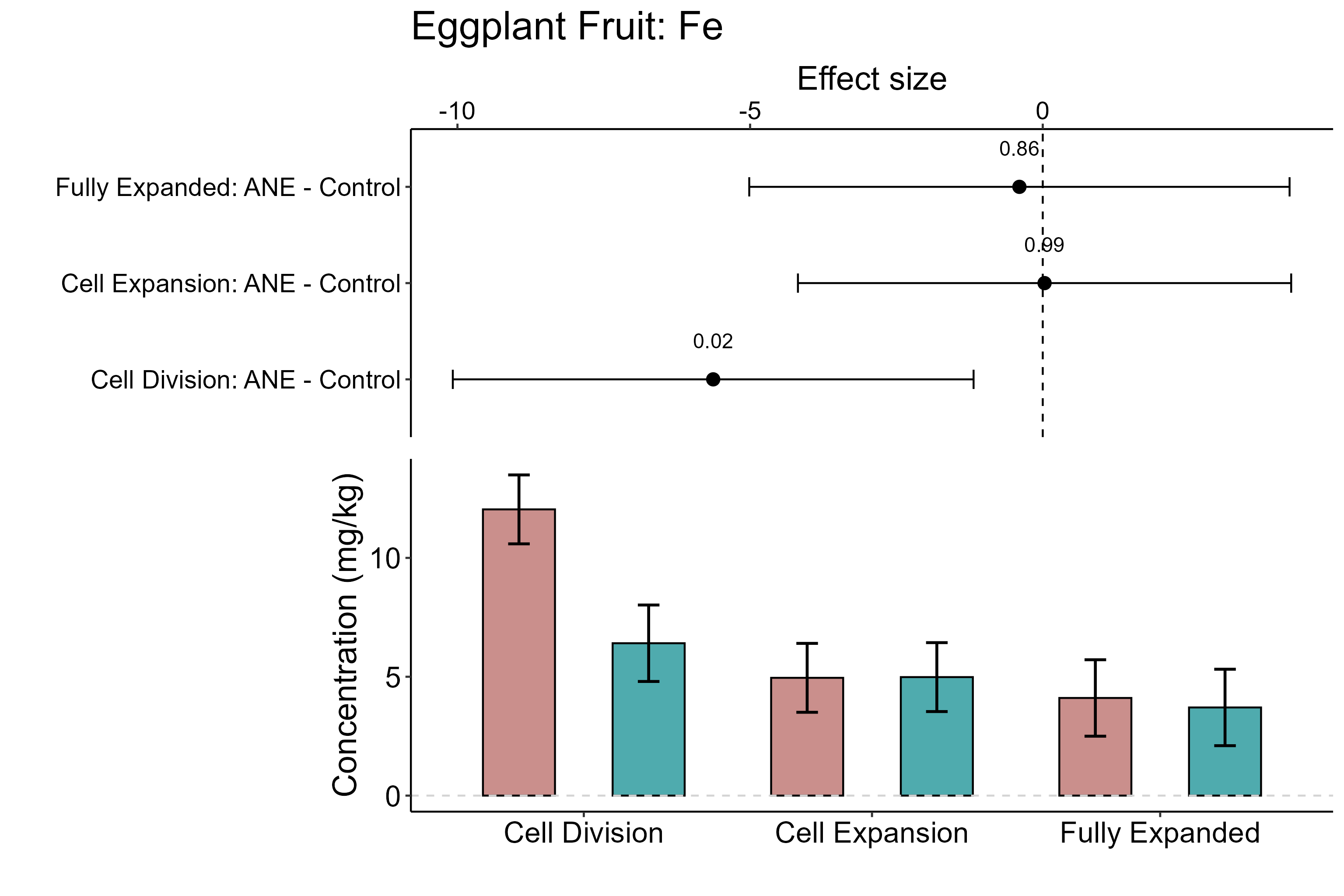

Supplement: Supplementary file 1 [file DataSheet1.zip › Micronutrients_barcharts/Eggplant_Fruit_Fe.png]

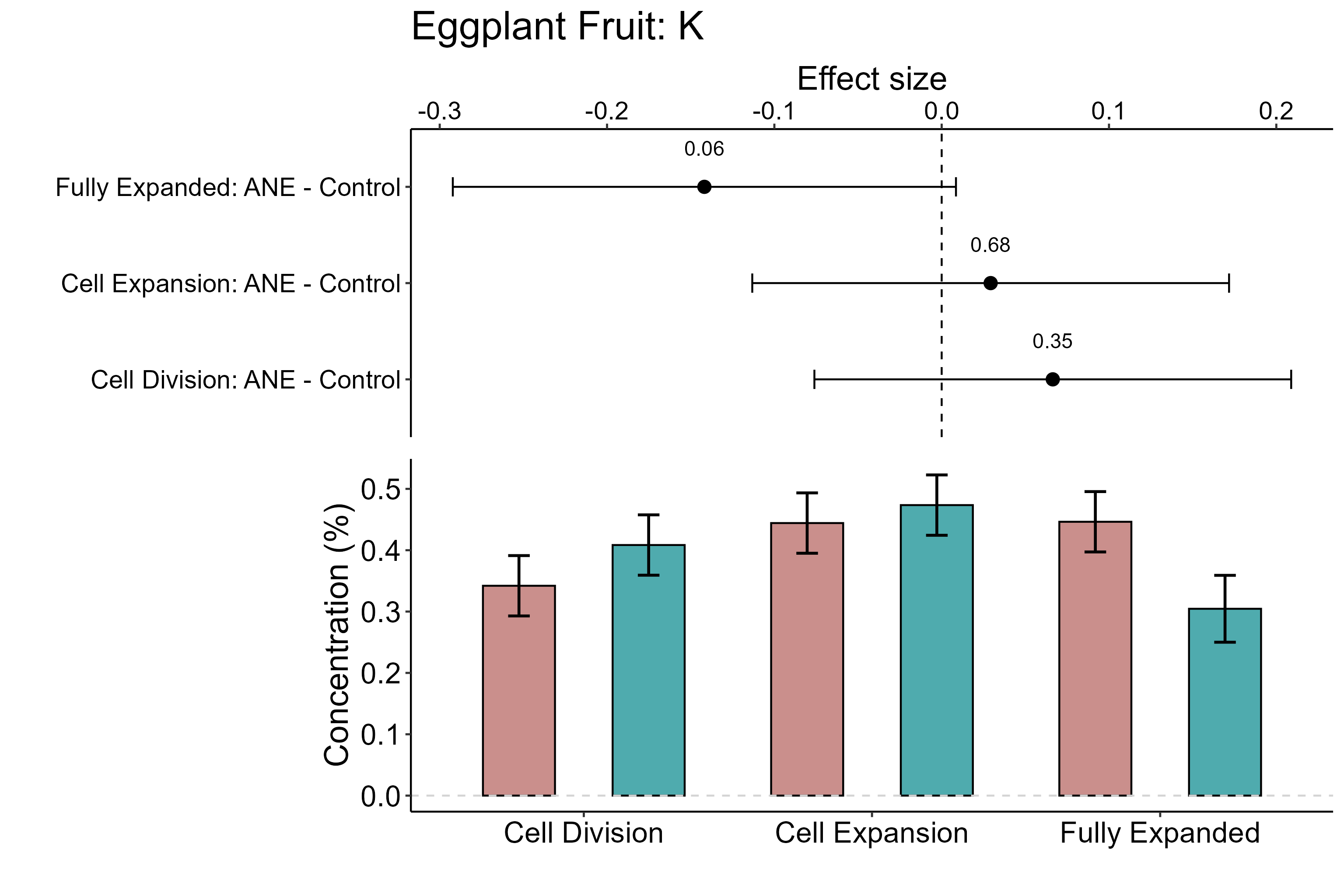

Supplement: Supplementary file 1 [file DataSheet1.zip › Micronutrients_barcharts/Eggplant_Fruit_K.png]

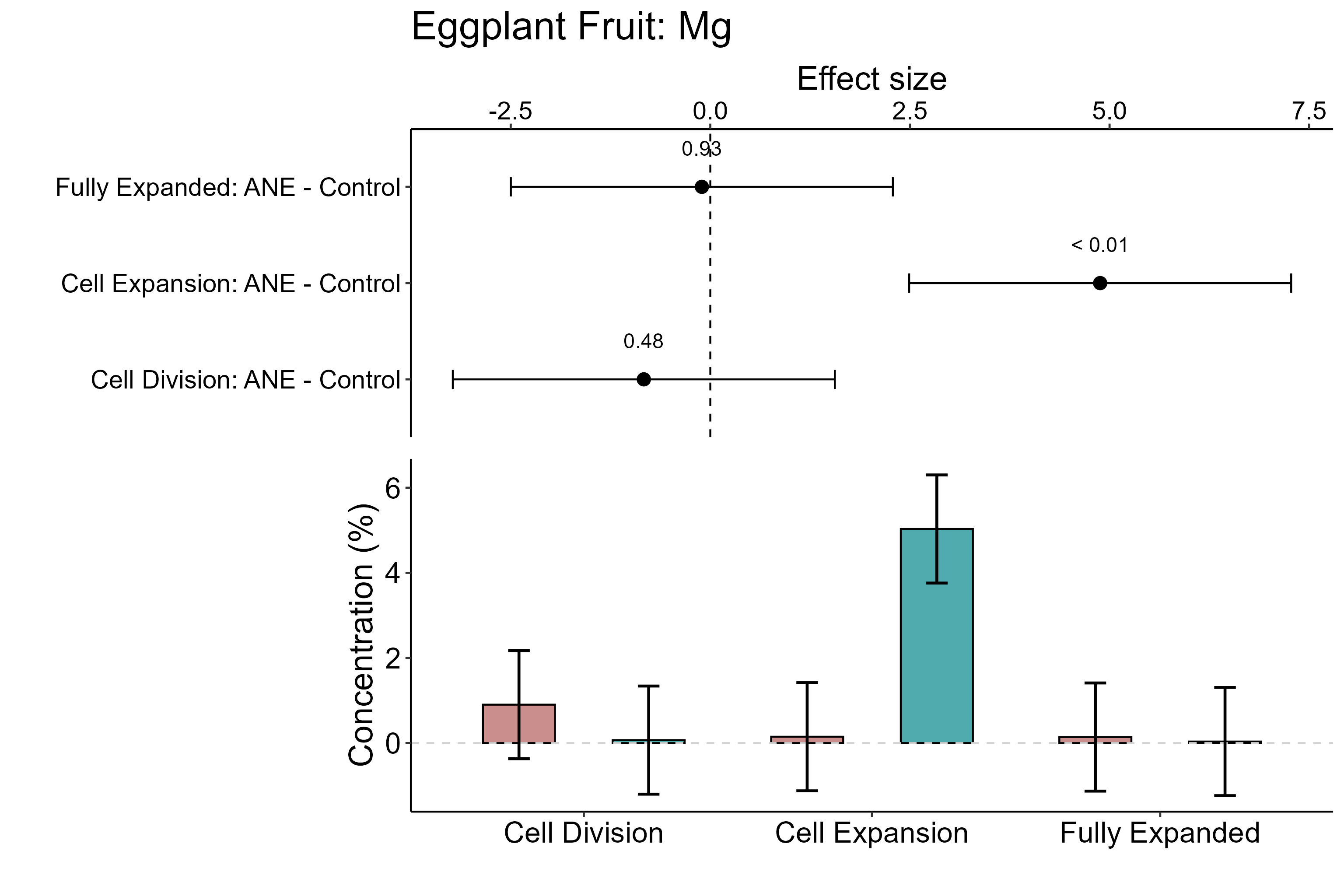

Supplement: Supplementary file 1 [file DataSheet1.zip › Micronutrients_barcharts/Eggplant_Fruit_Mg.png]

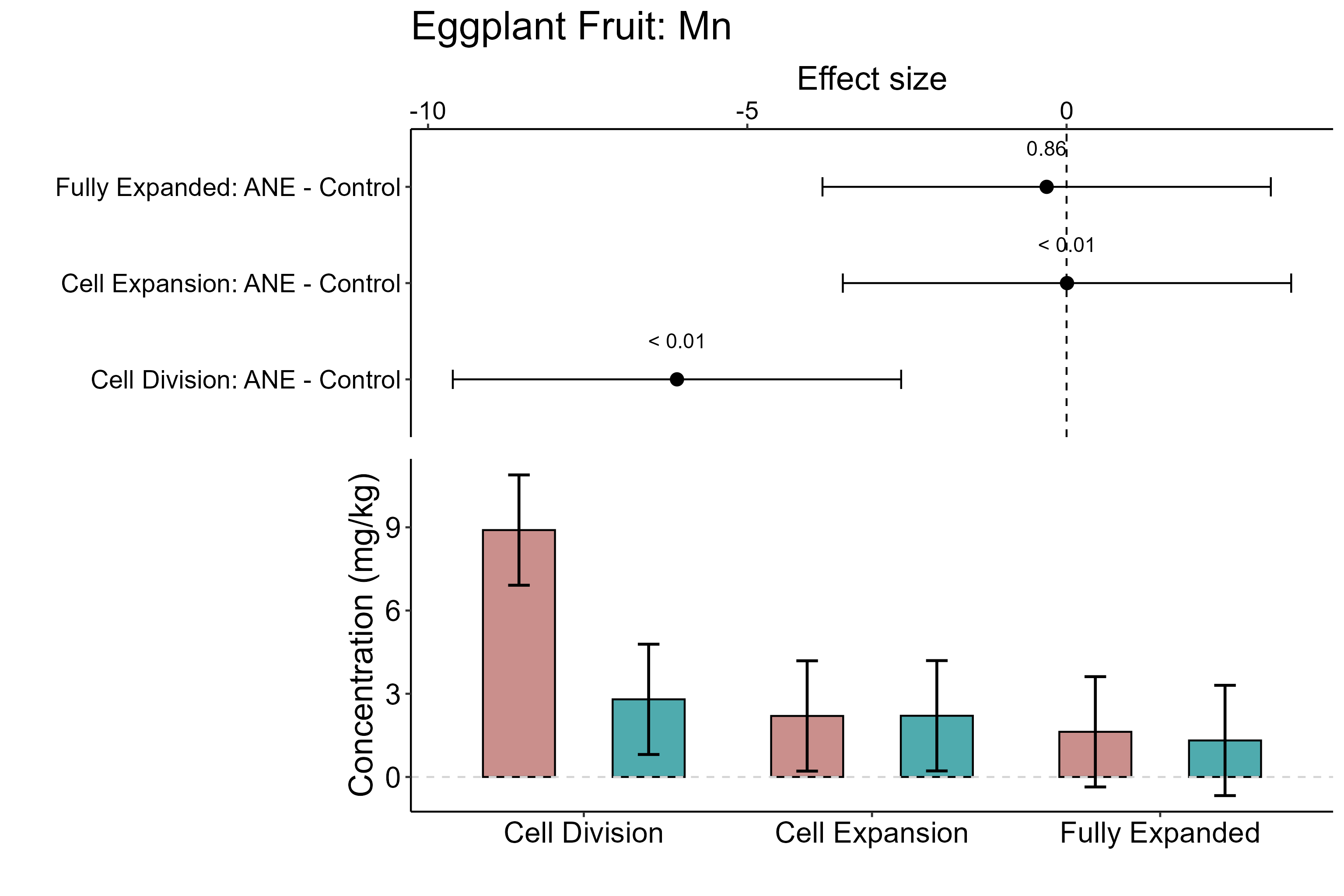

Supplement: Supplementary file 1 [file DataSheet1.zip › Micronutrients_barcharts/Eggplant_Fruit_Mn.png]

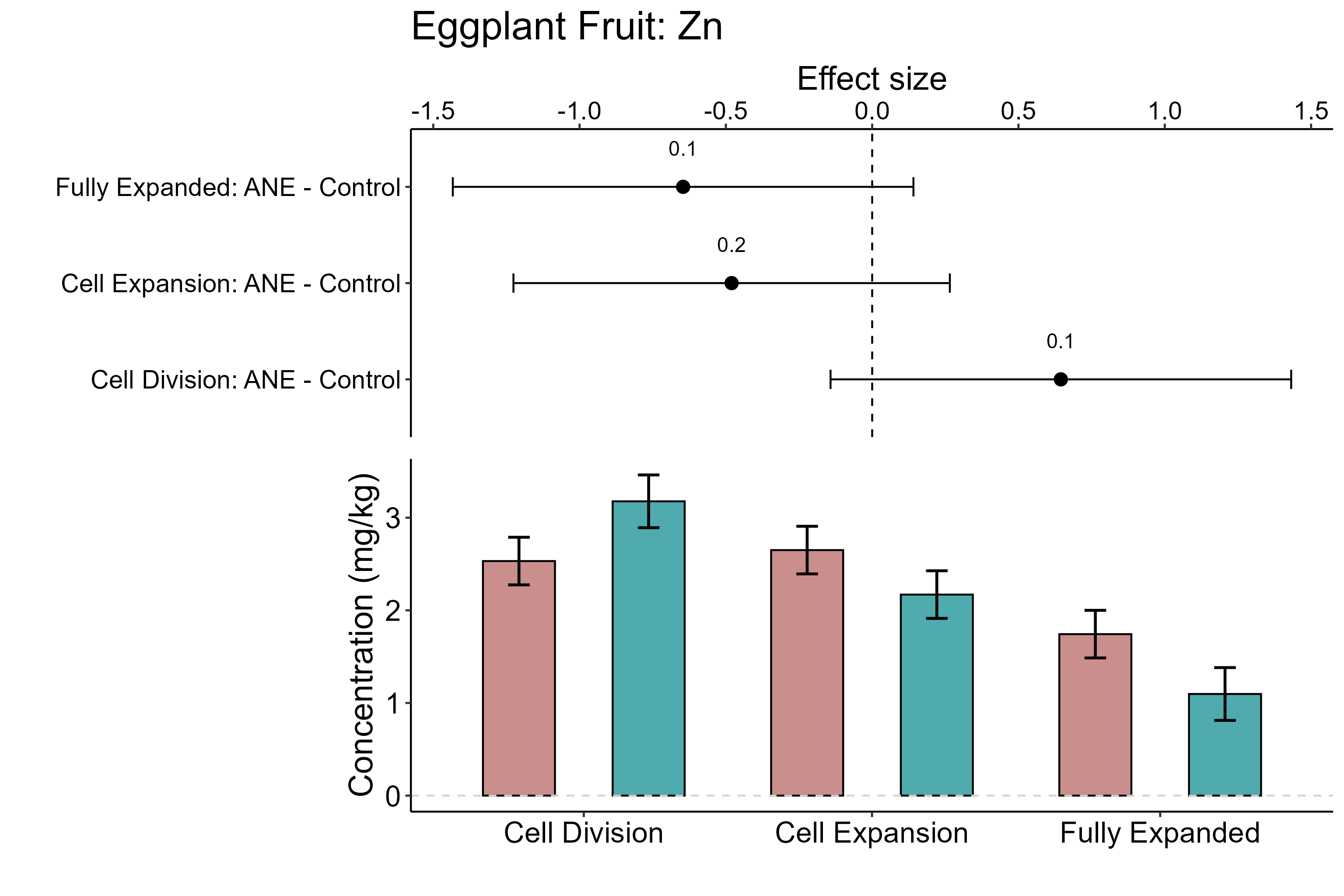

Supplement: Supplementary file 1 [file DataSheet1.zip › Micronutrients_barcharts/Eggplant_Fruit_Zn.png]

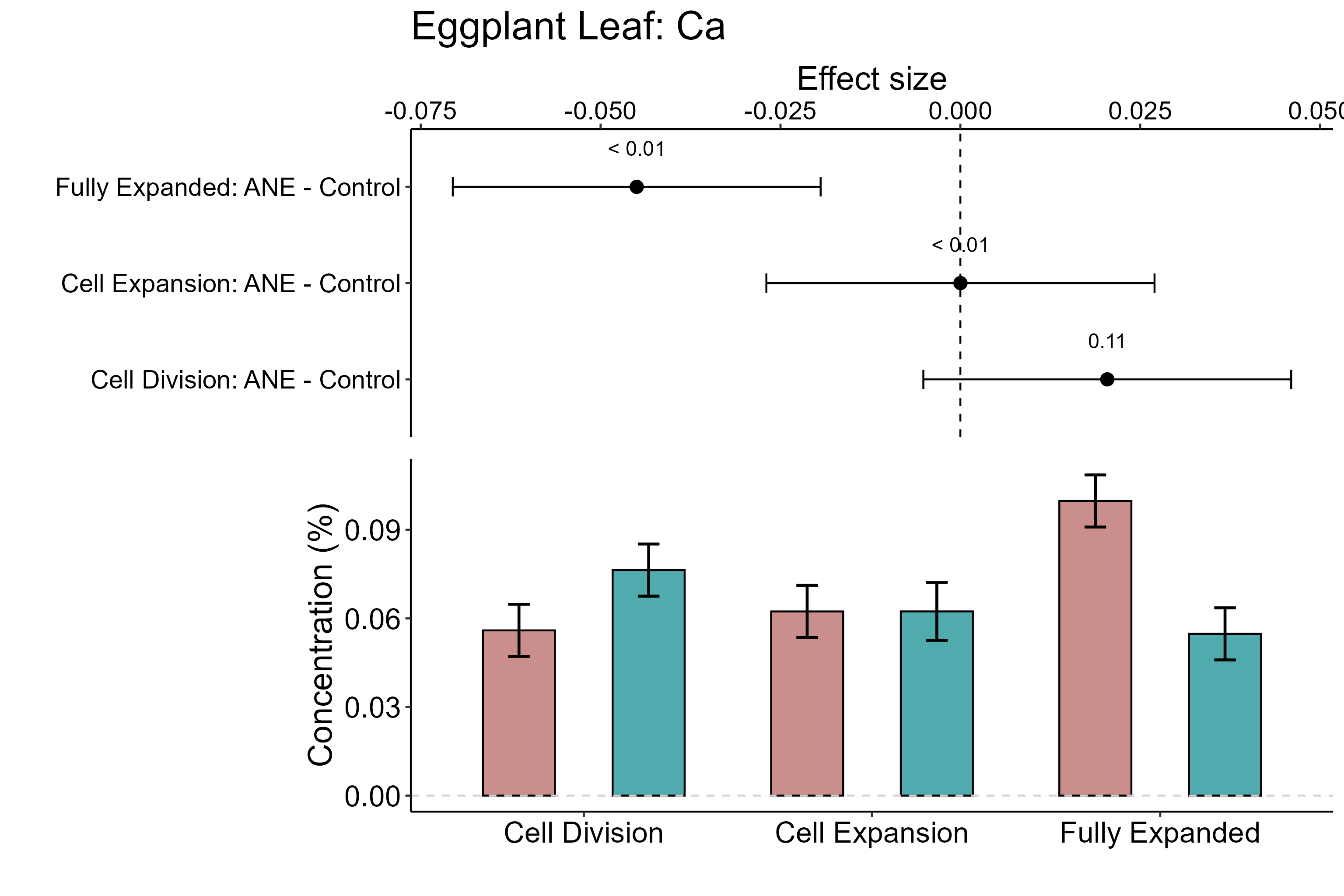

Supplement: Supplementary file 1 [file DataSheet1.zip › Micronutrients_barcharts/Eggplant_Leaf_Ca.png]

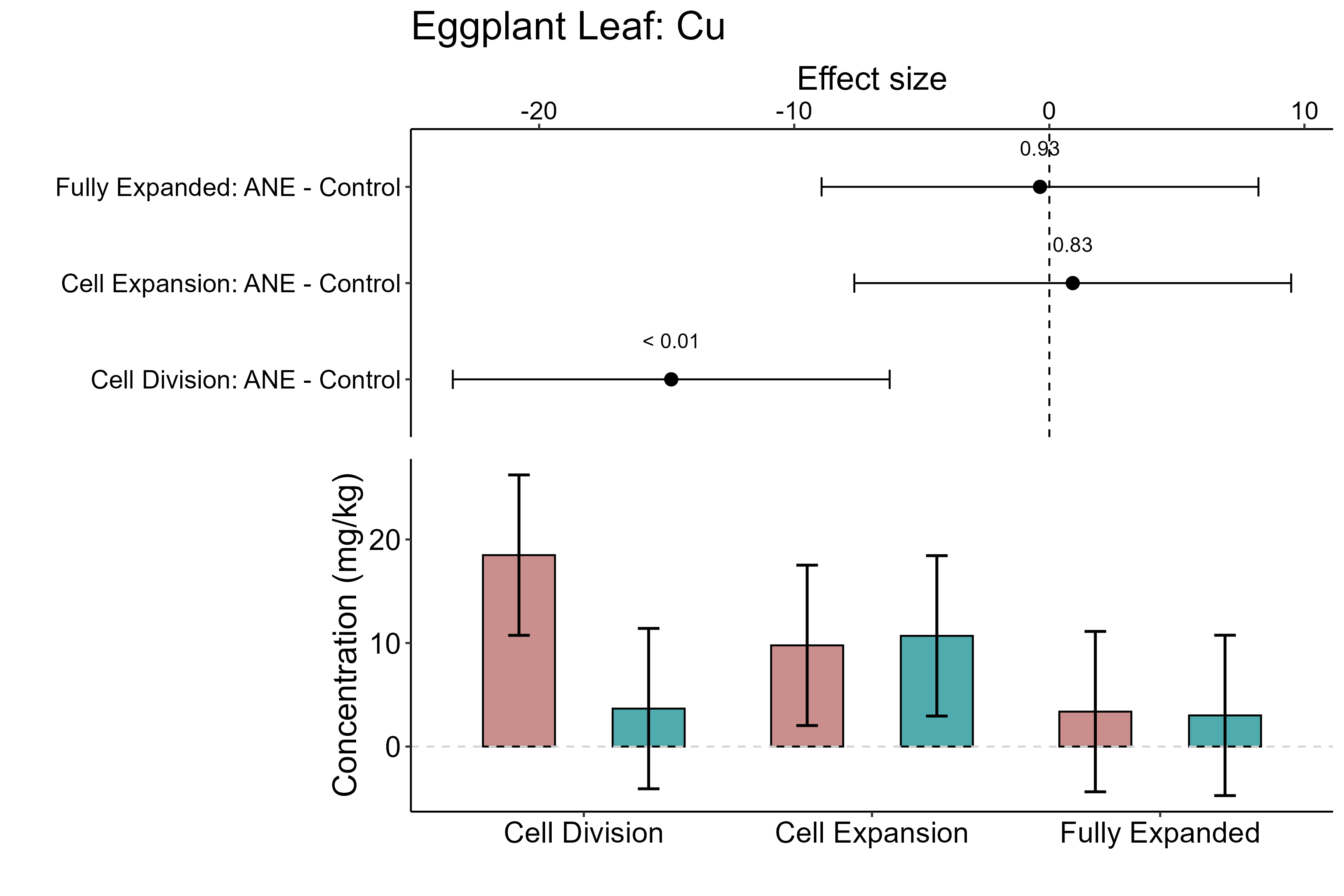

Supplement: Supplementary file 1 [file DataSheet1.zip › Micronutrients_barcharts/Eggplant_Leaf_Cu.png]

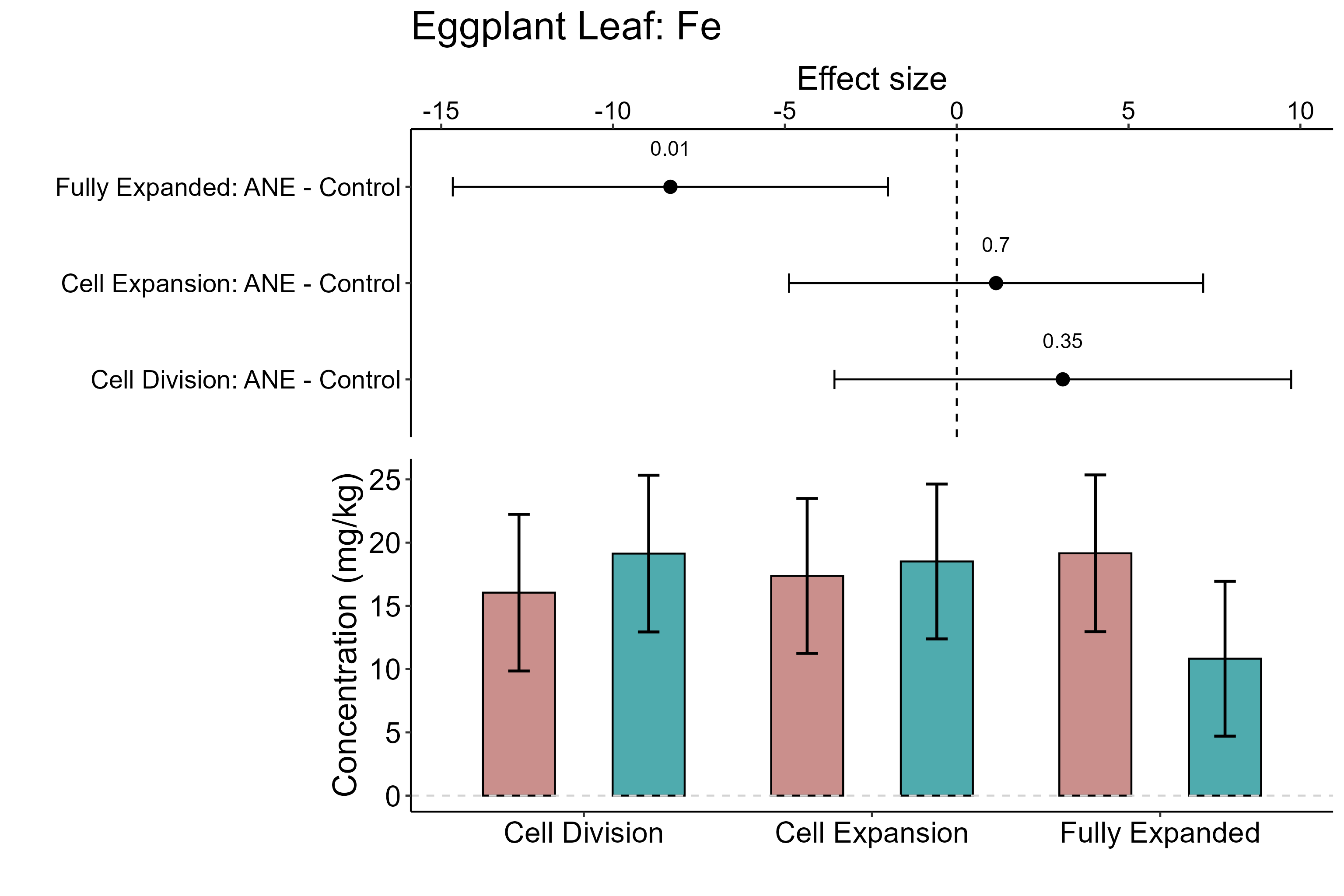

Supplement: Supplementary file 1 [file DataSheet1.zip › Micronutrients_barcharts/Eggplant_Leaf_Fe.png]

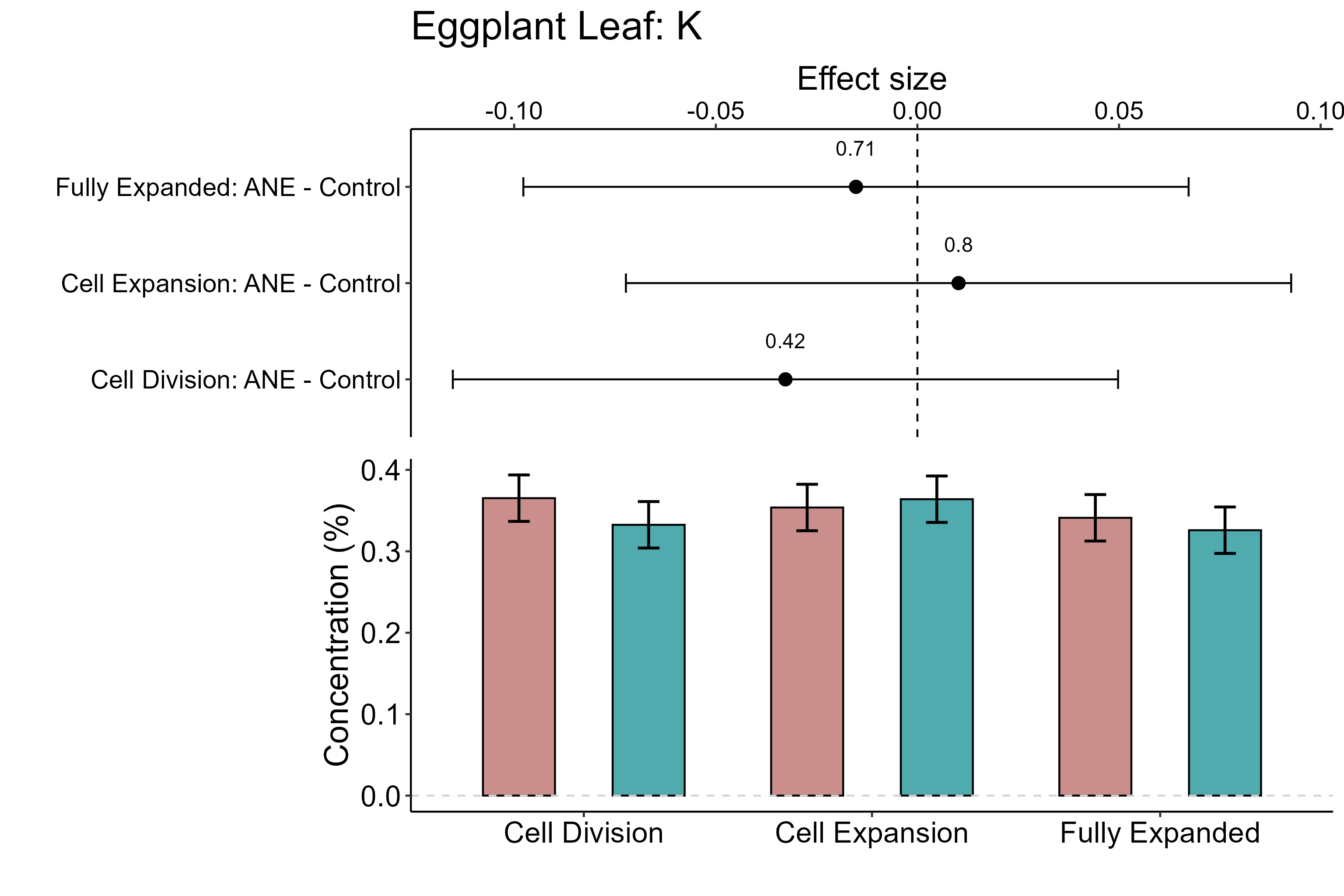

Supplement: Supplementary file 1 [file DataSheet1.zip › Micronutrients_barcharts/Eggplant_Leaf_K.png]

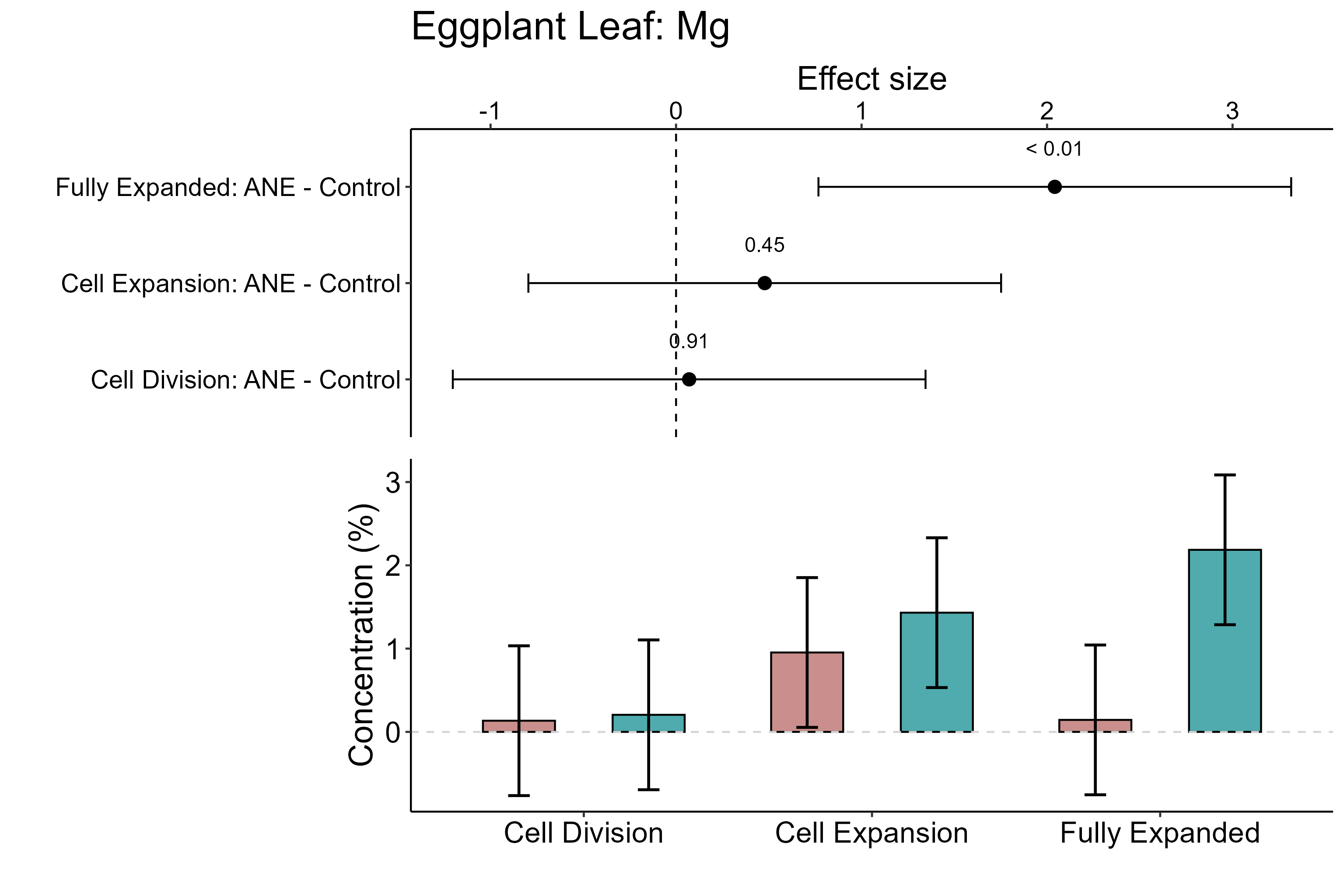

Supplement: Supplementary file 1 [file DataSheet1.zip › Micronutrients_barcharts/Eggplant_Leaf_Mg.png]

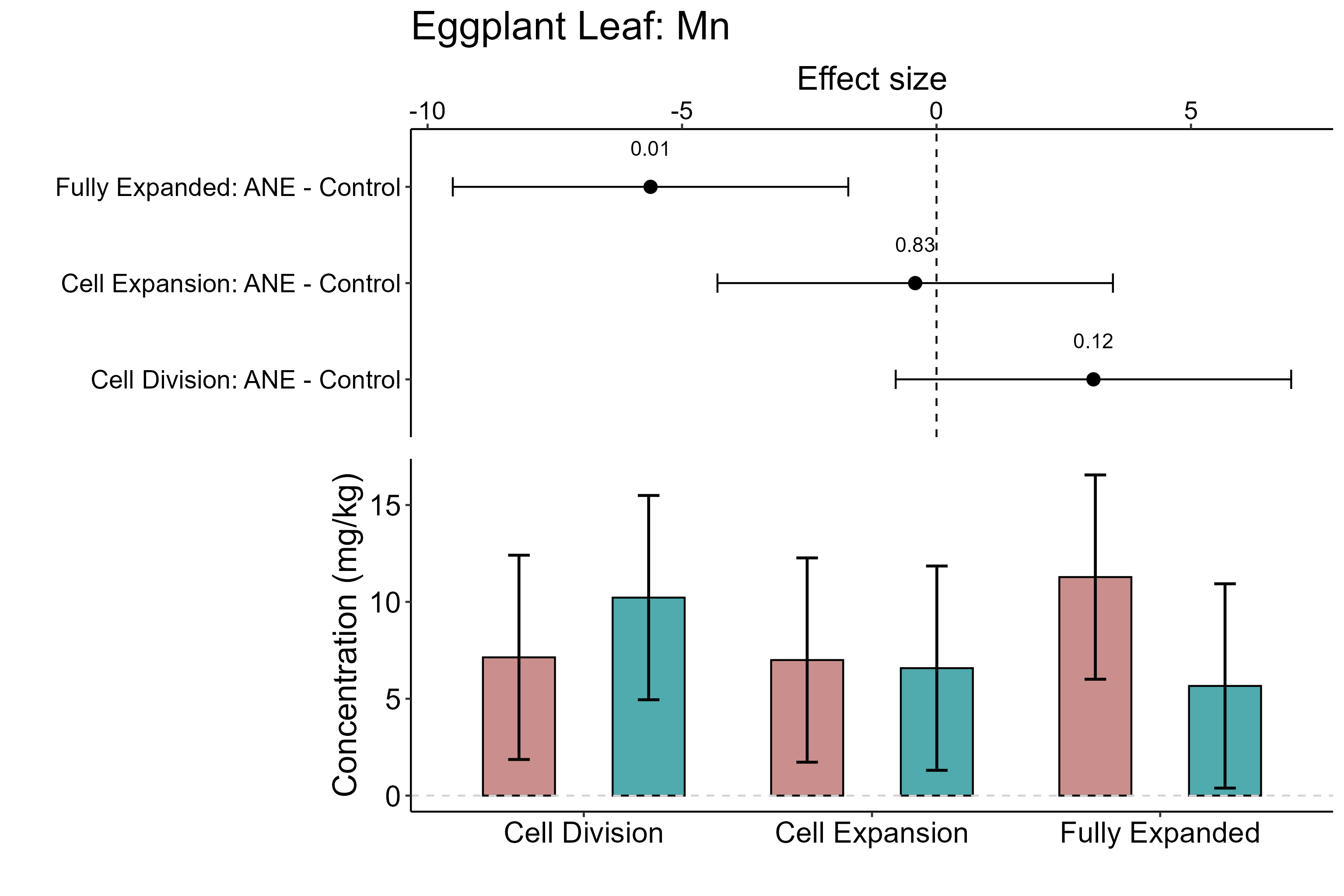

Supplement: Supplementary file 1 [file DataSheet1.zip › Micronutrients_barcharts/Eggplant_Leaf_Mn.png]

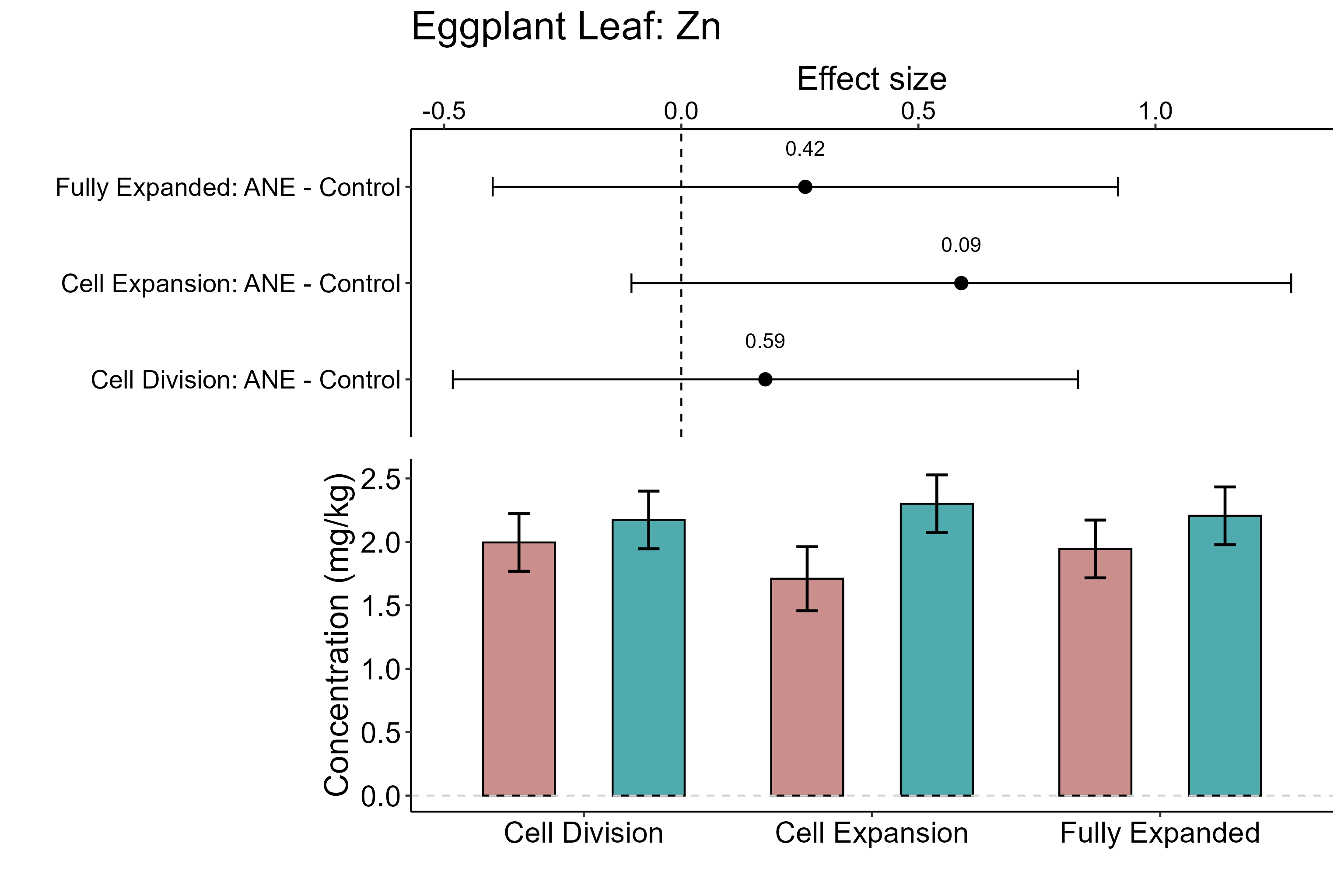

Supplement: Supplementary file 1 [file DataSheet1.zip › Micronutrients_barcharts/Eggplant_Leaf_Zn.png]

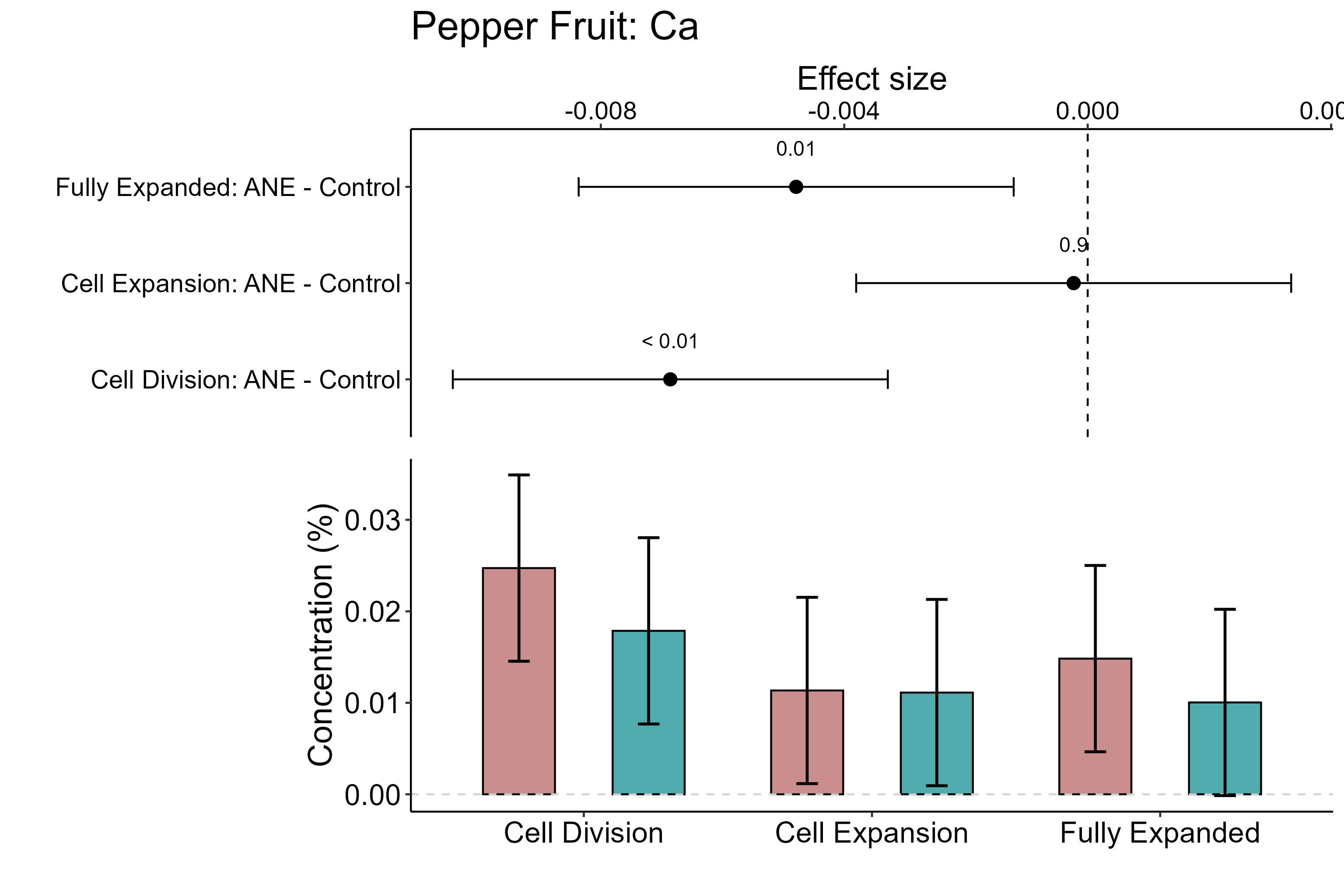

Supplement: Supplementary file 1 [file DataSheet1.zip › Micronutrients_barcharts/Pepper_Fruit_Ca.png]

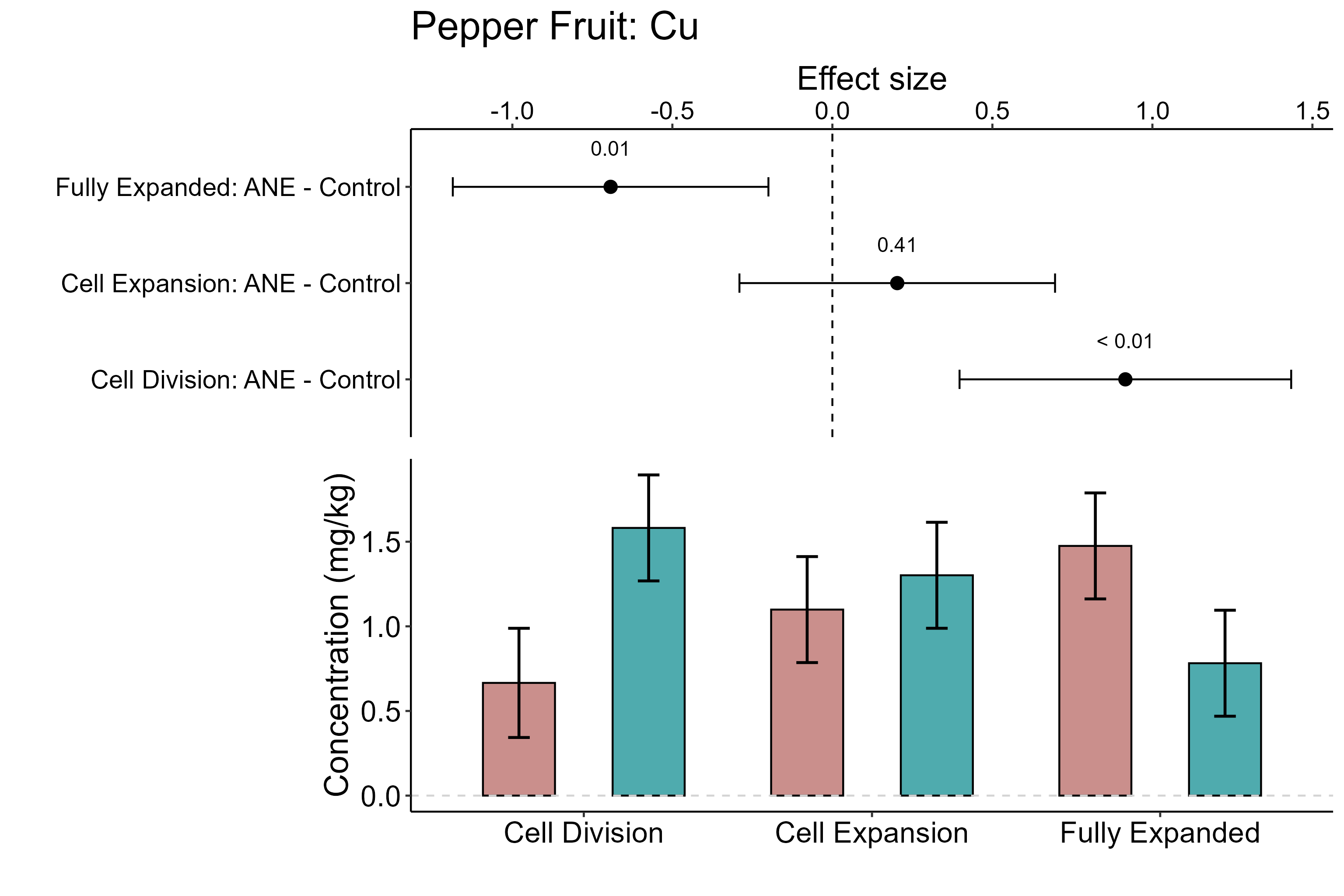

Supplement: Supplementary file 1 [file DataSheet1.zip › Micronutrients_barcharts/Pepper_Fruit_Cu.png]

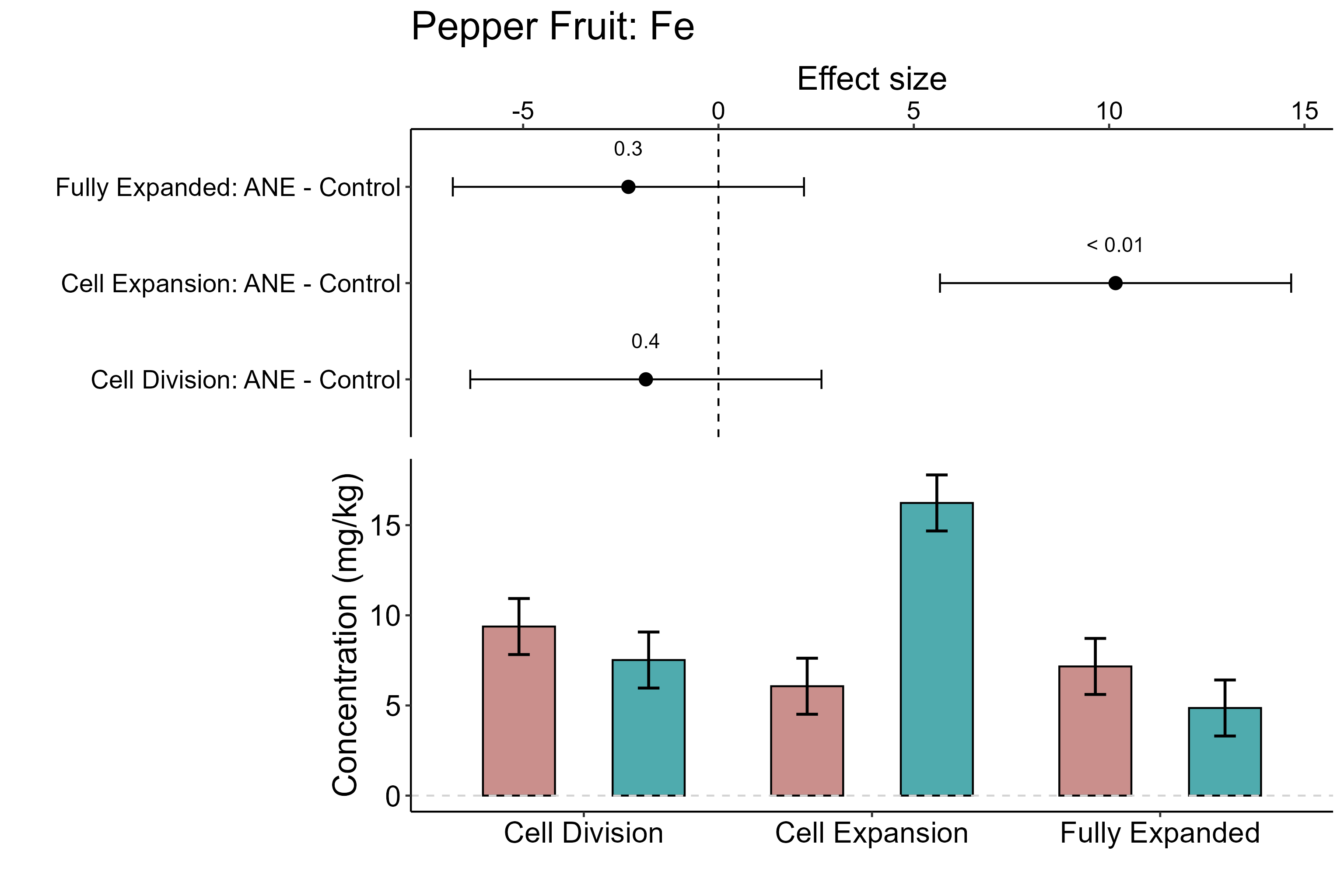

Supplement: Supplementary file 1 [file DataSheet1.zip › Micronutrients_barcharts/Pepper_Fruit_Fe.png]

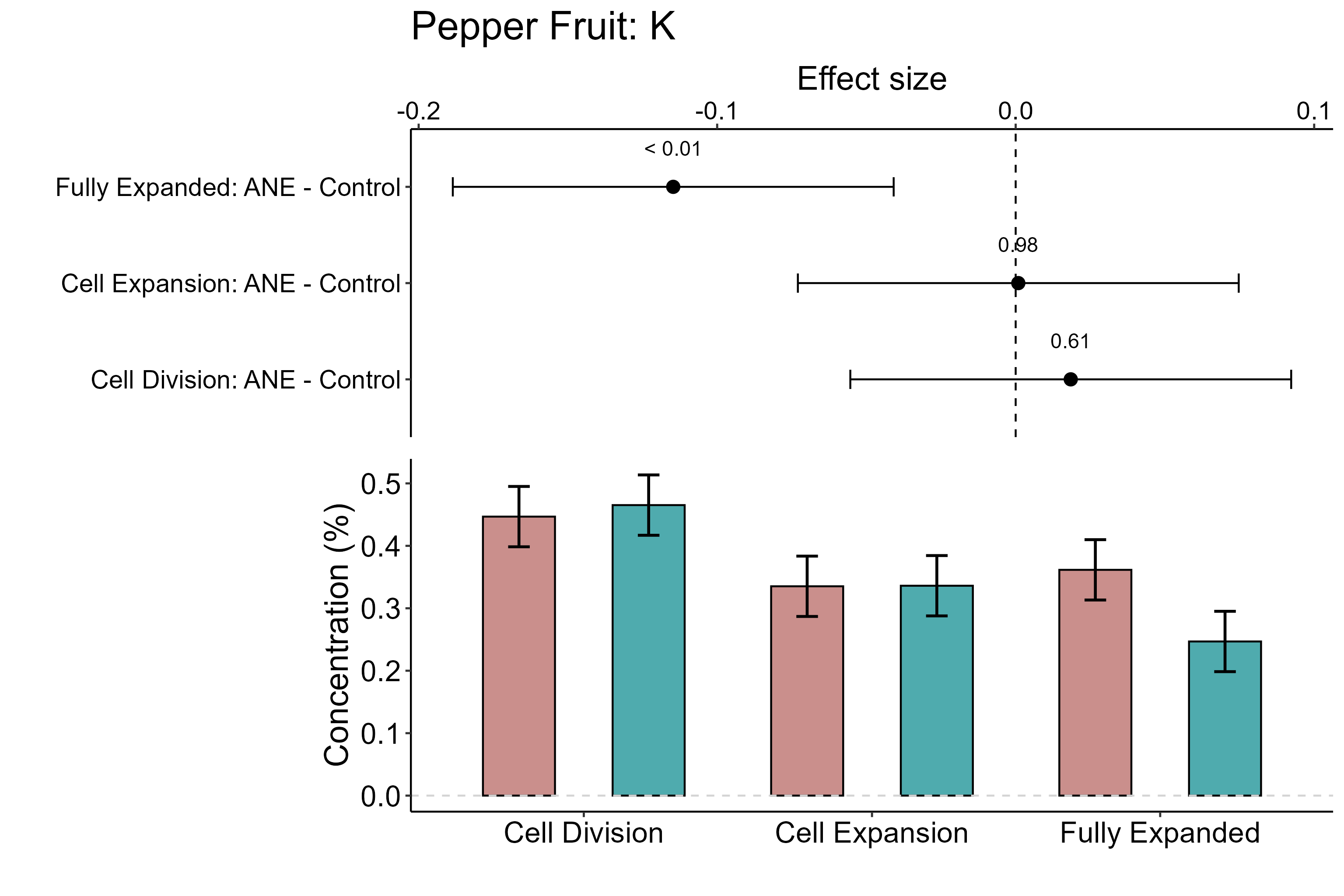

Supplement: Supplementary file 1 [file DataSheet1.zip › Micronutrients_barcharts/Pepper_Fruit_K.png]

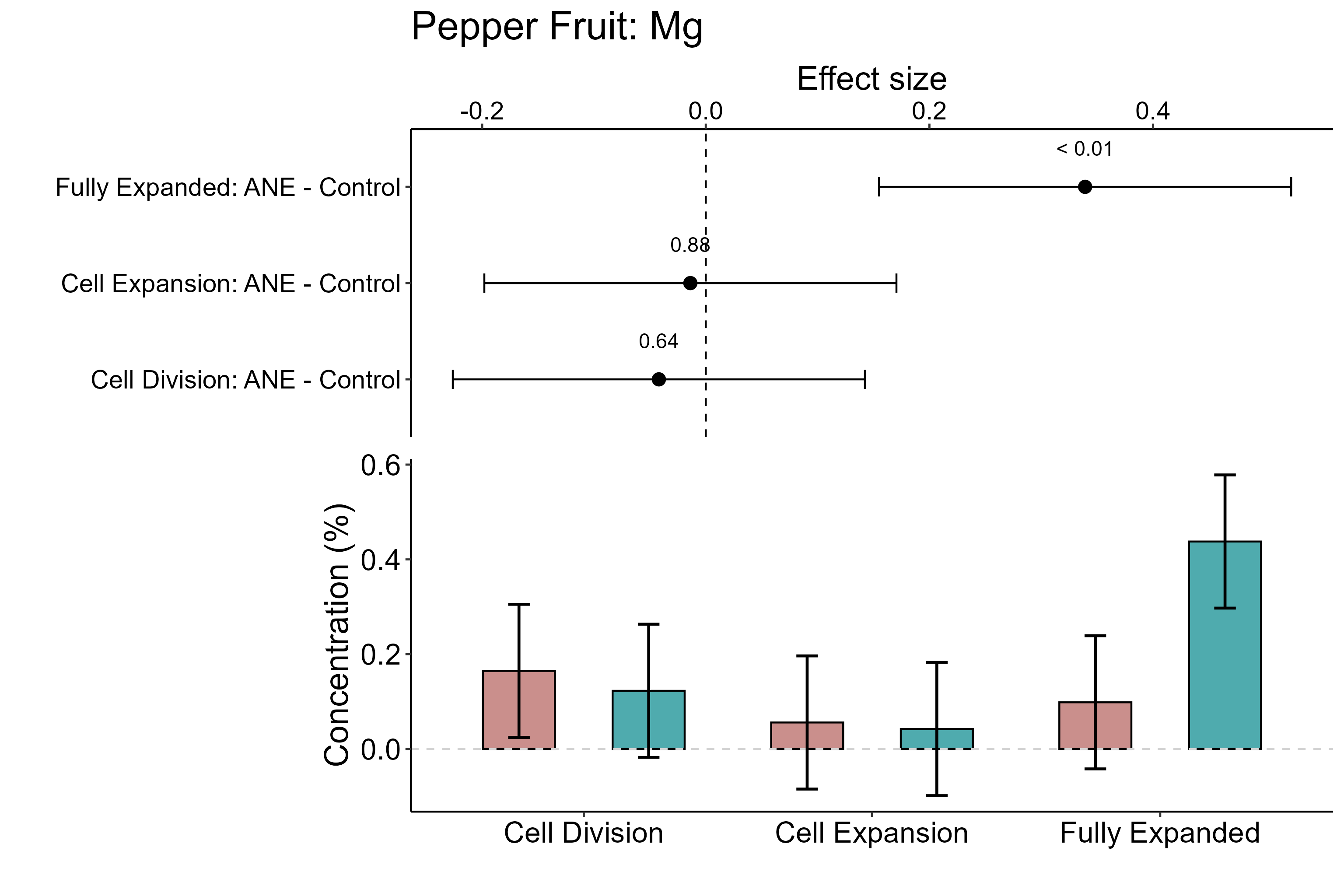

Supplement: Supplementary file 1 [file DataSheet1.zip › Micronutrients_barcharts/Pepper_Fruit_Mg.png]

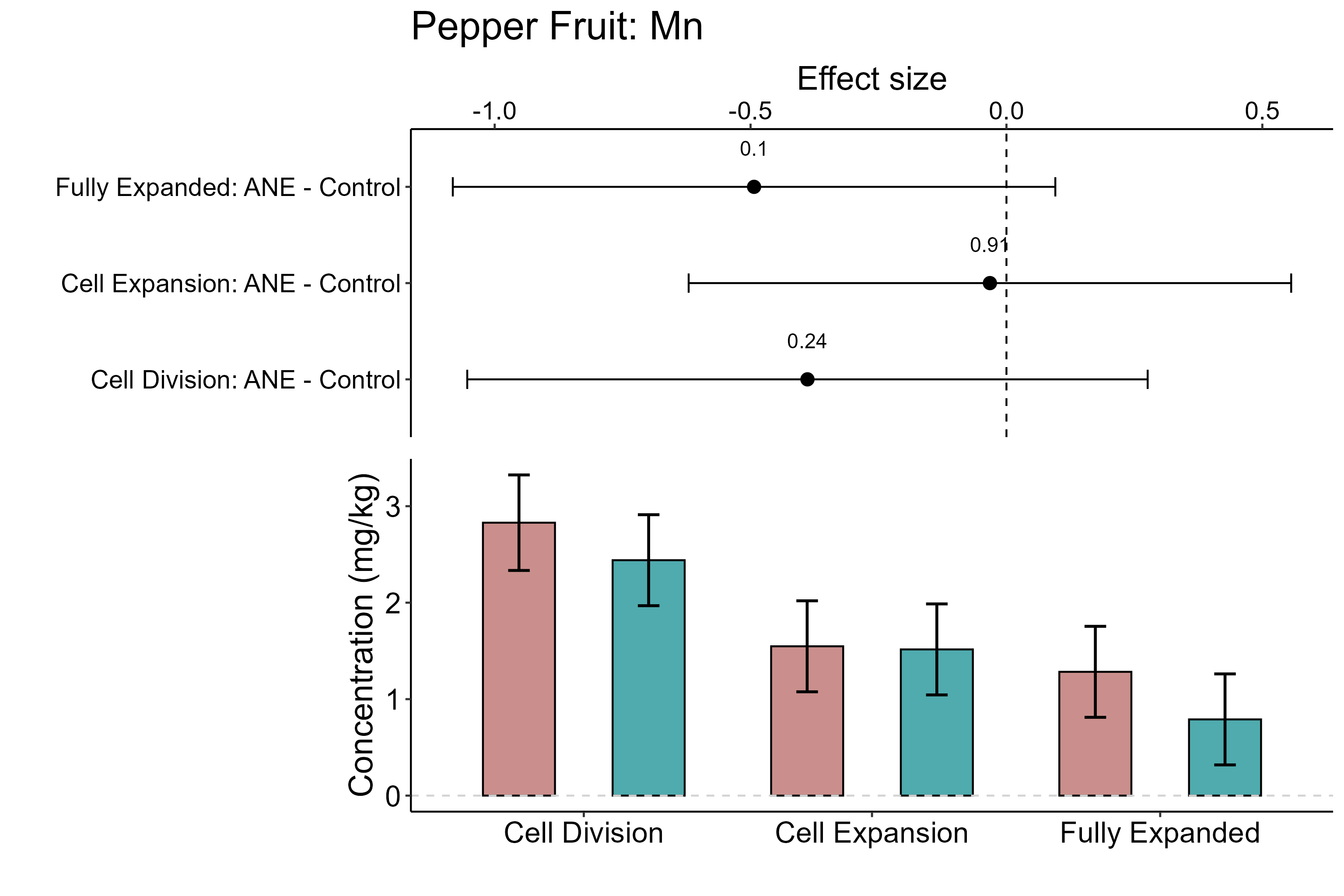

Supplement: Supplementary file 1 [file DataSheet1.zip › Micronutrients_barcharts/Pepper_Fruit_Mn.png]

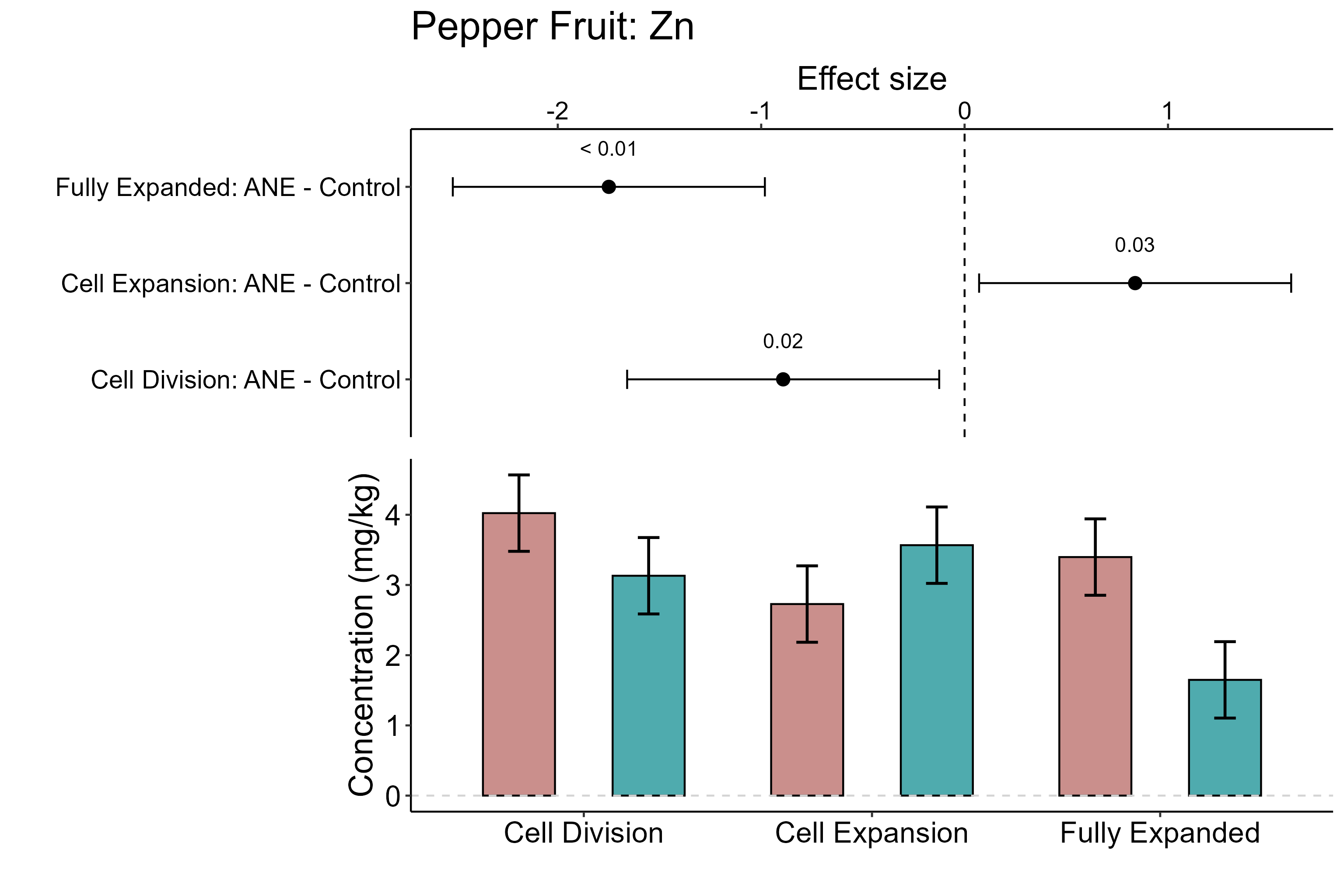

Supplement: Supplementary file 1 [file DataSheet1.zip › Micronutrients_barcharts/Pepper_Fruit_Zn.png]

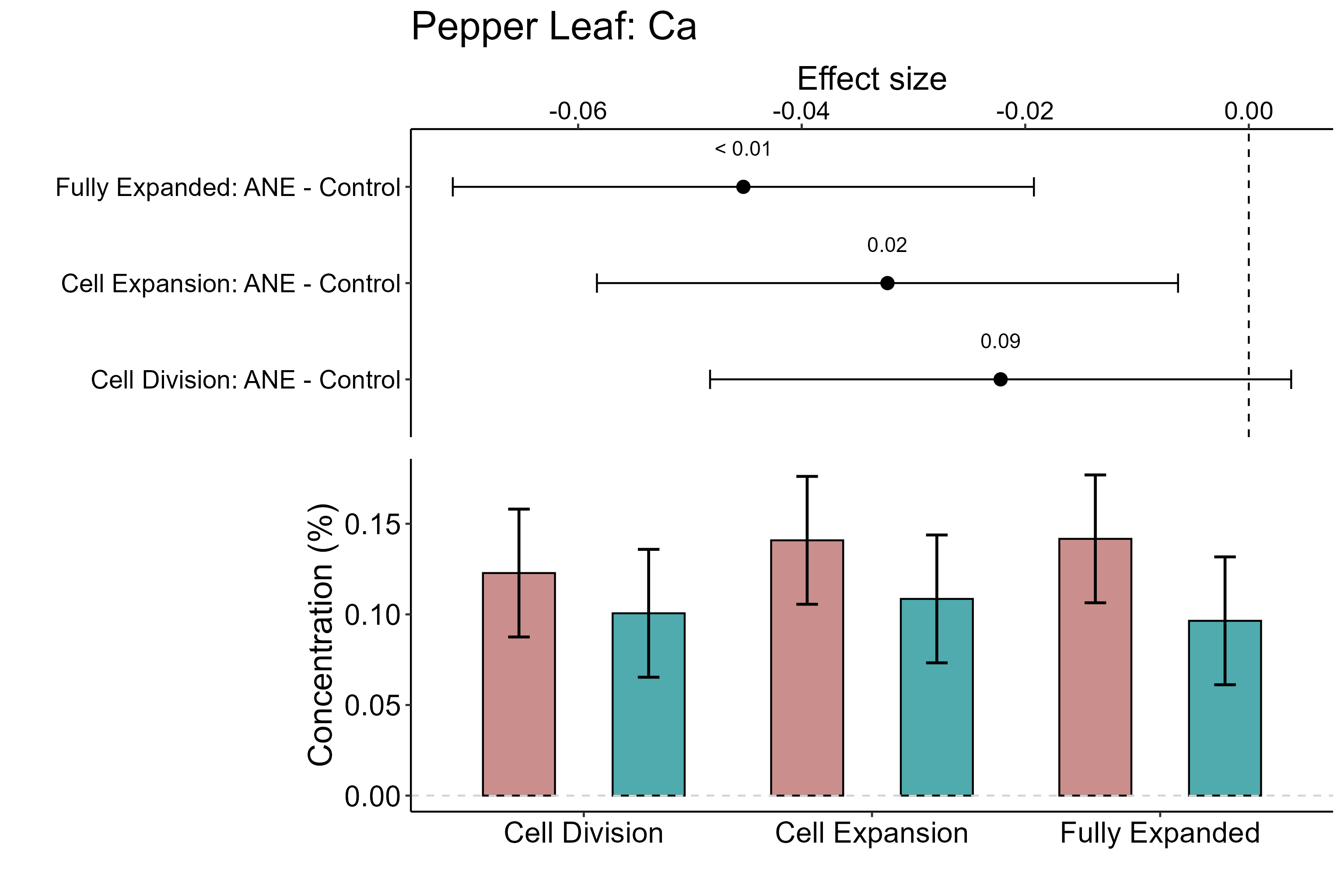

Supplement: Supplementary file 1 [file DataSheet1.zip › Micronutrients_barcharts/Pepper_Leaf_Ca.png]

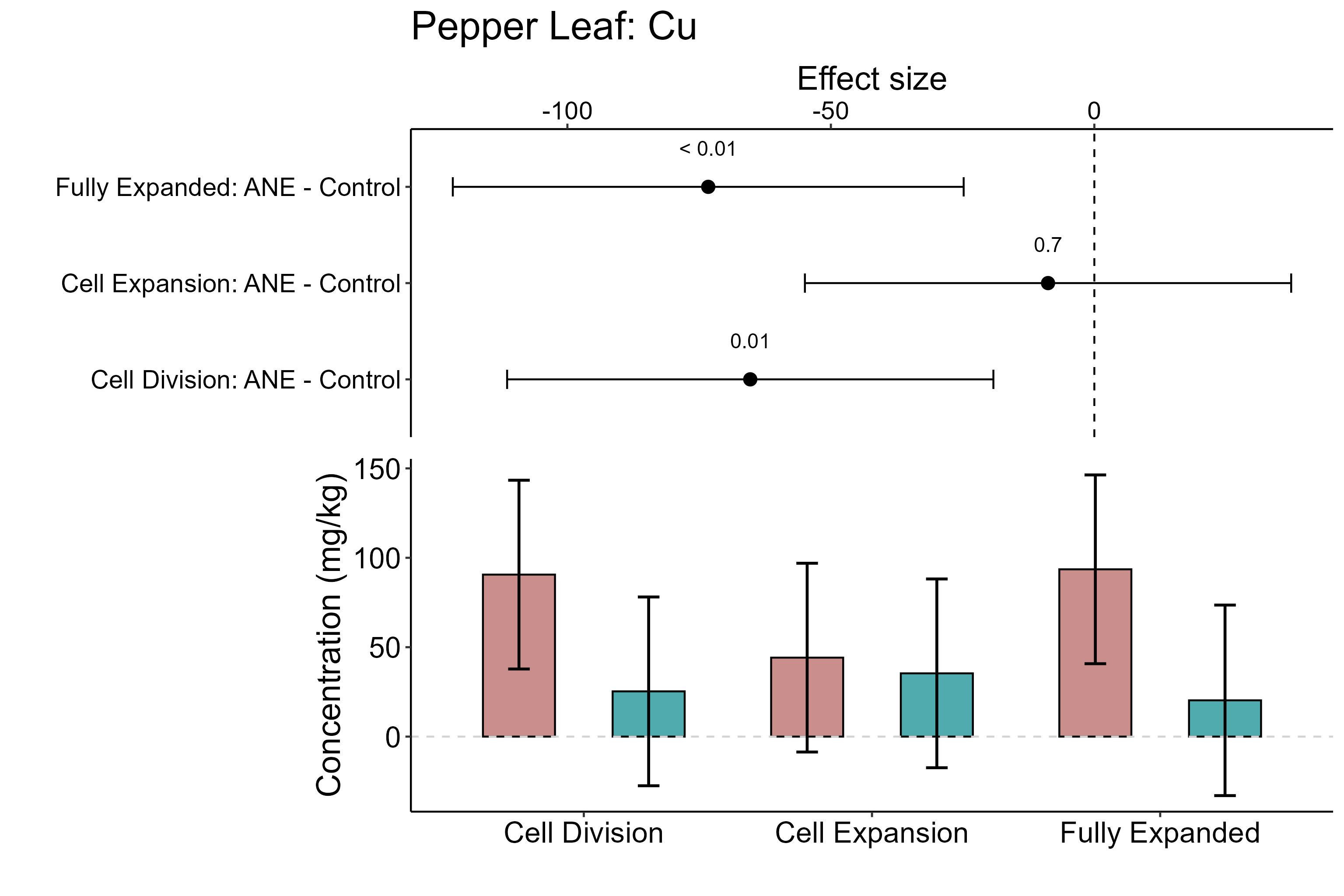

Supplement: Supplementary file 1 [file DataSheet1.zip › Micronutrients_barcharts/Pepper_Leaf_Cu.png]

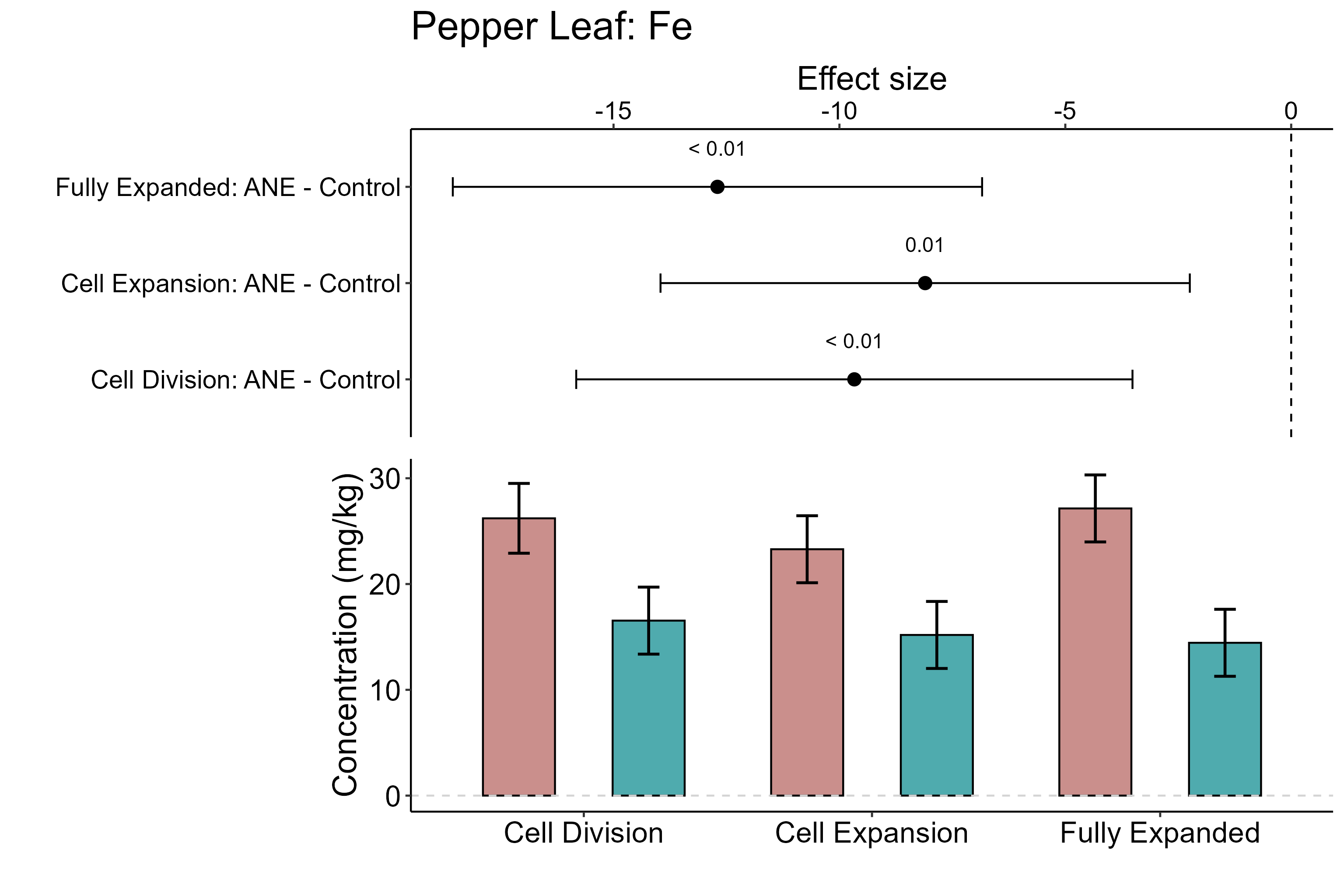

Supplement: Supplementary file 1 [file DataSheet1.zip › Micronutrients_barcharts/Pepper_Leaf_Fe.png]

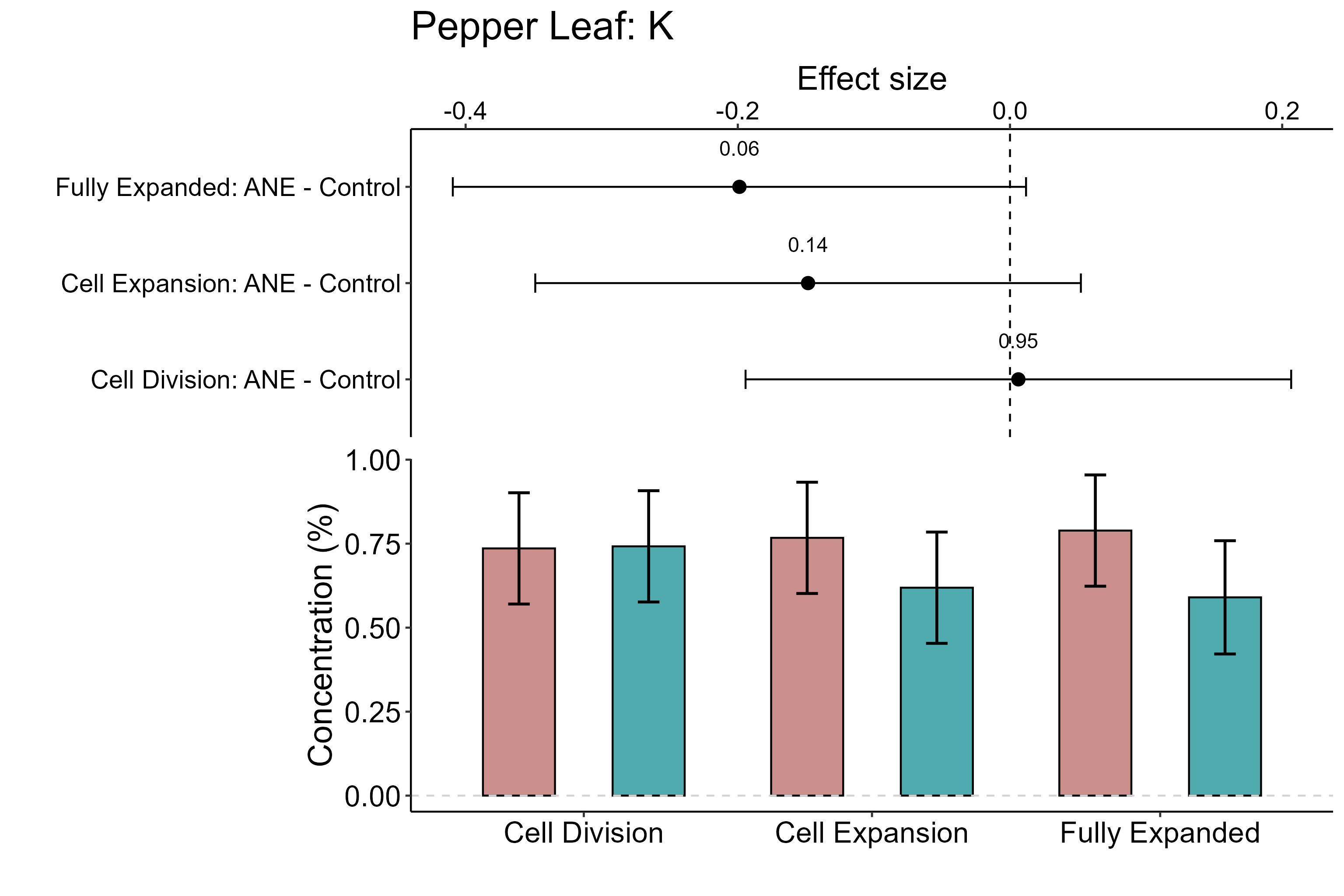

Supplement: Supplementary file 1 [file DataSheet1.zip › Micronutrients_barcharts/Pepper_Leaf_K.png]

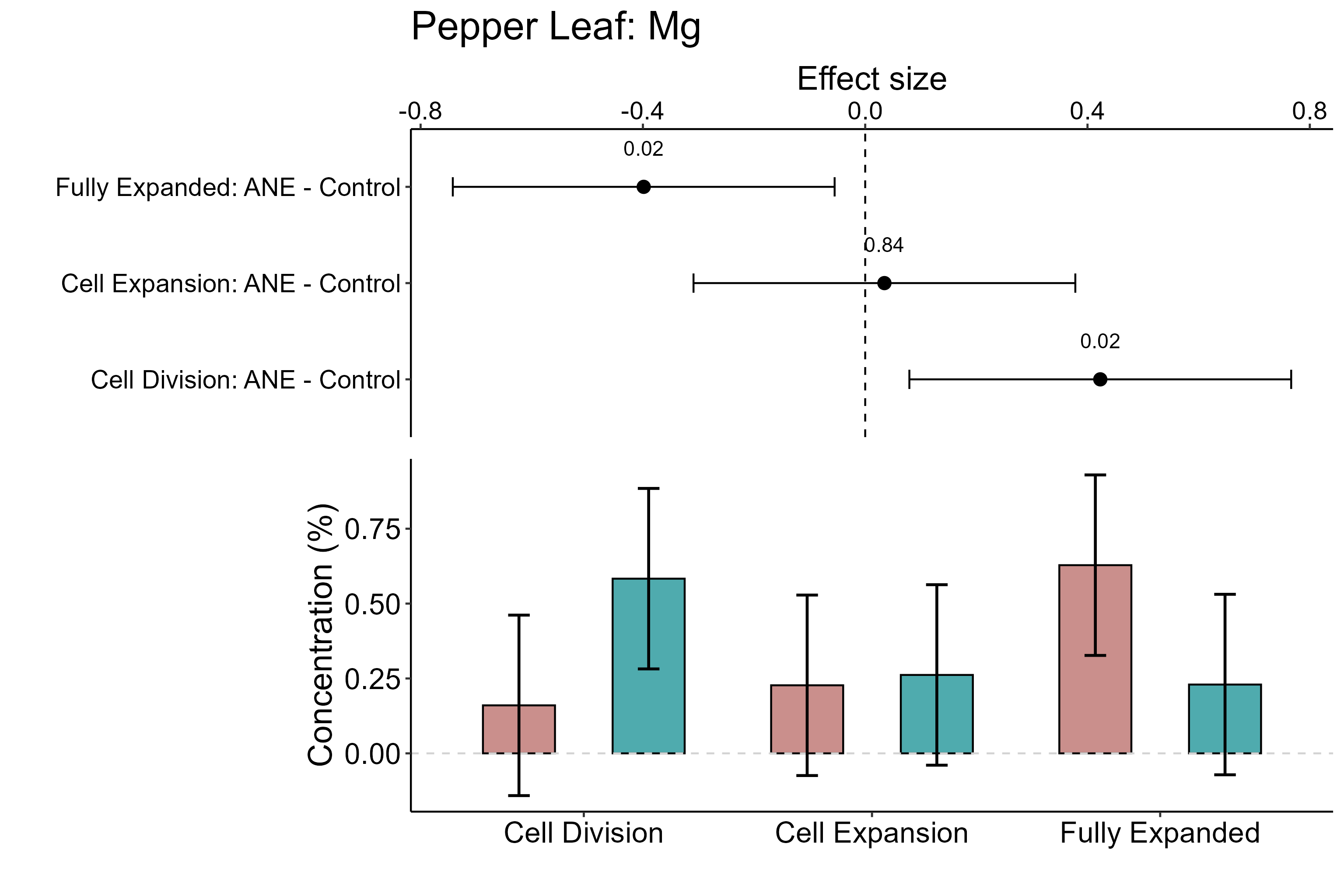

Supplement: Supplementary file 1 [file DataSheet1.zip › Micronutrients_barcharts/Pepper_Leaf_Mg.png]

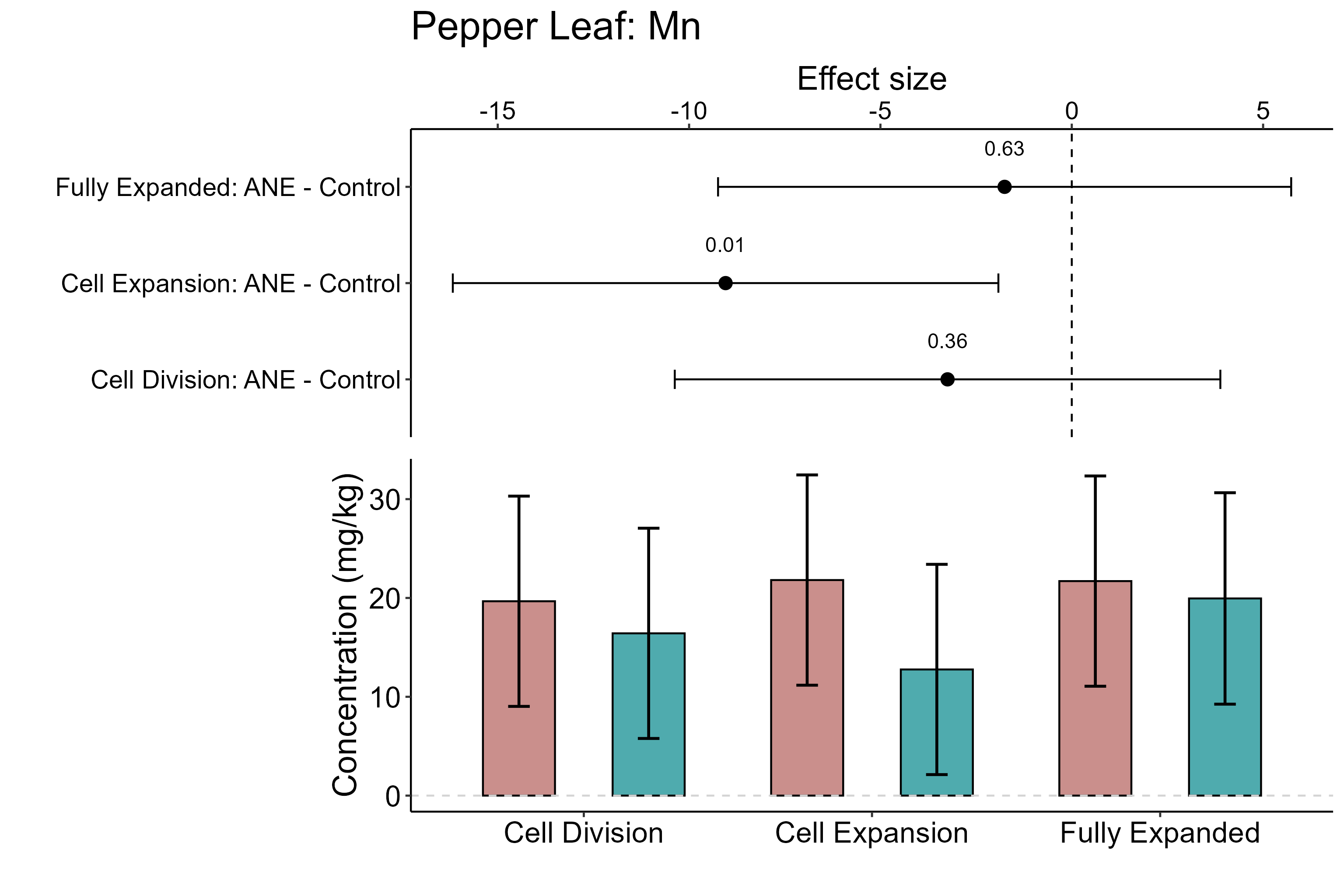

Supplement: Supplementary file 1 [file DataSheet1.zip › Micronutrients_barcharts/Pepper_Leaf_Mn.png]

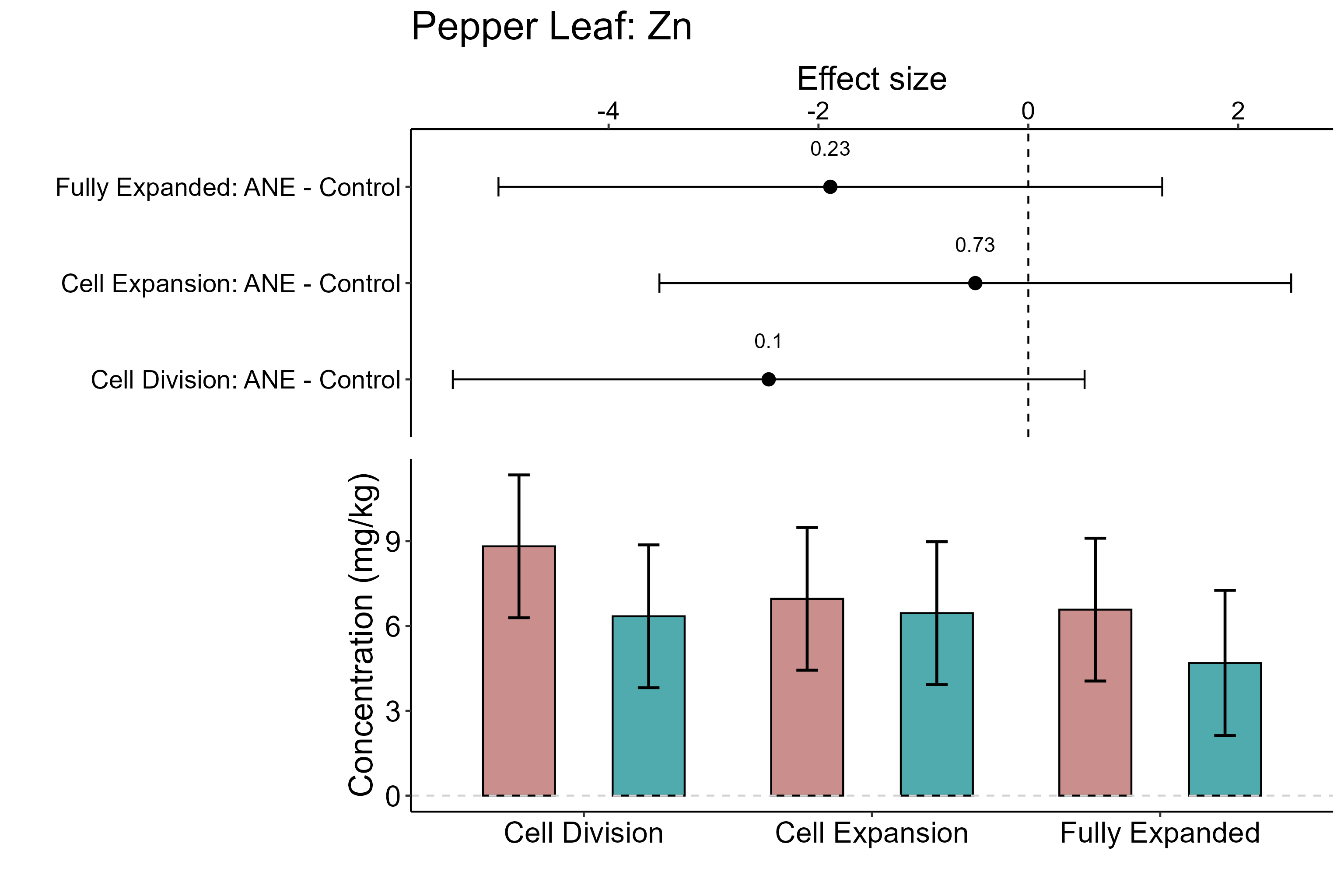

Supplement: Supplementary file 1 [file DataSheet1.zip › Micronutrients_barcharts/Pepper_Leaf_Zn.png]

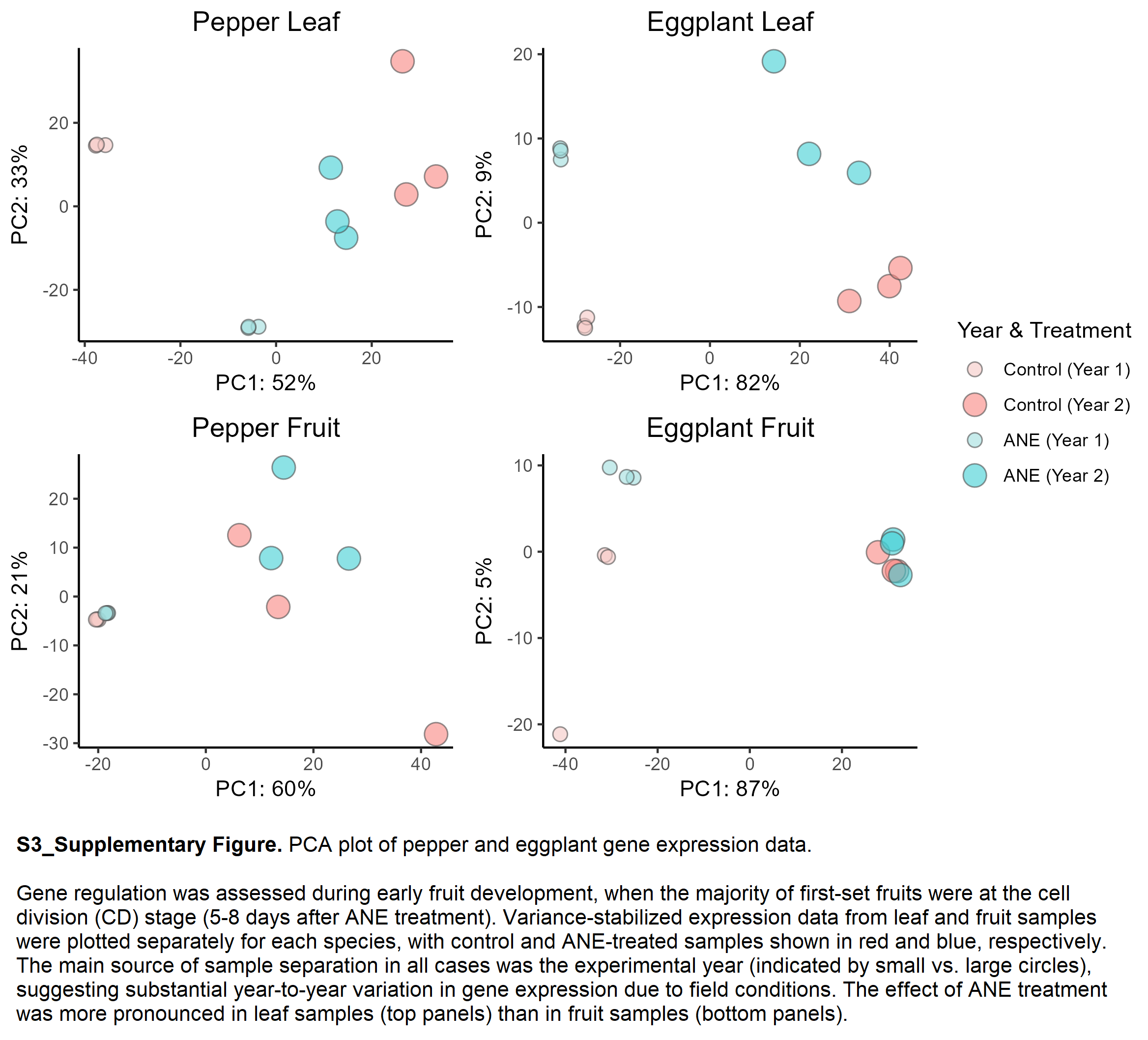

Supplement: Supplementary file 2 [file Image1.png]
